# Lipid Profiles of Urinary Extracellular Vesicles Released during the Inactive and Active Phases of Aged Male Mice with Spontaneous Hypertension

**DOI:** 10.3390/ijms232315397

**Published:** 2022-12-06

**Authors:** Juliana Pena Lopez, Mohammad-Zaman Nouri, Areej Ebrahim, Kevin M. Chacko, Whitney C. Schramm, Mohammed F. Gholam, Tezcan Ozrazgat-Baslanti, Nancy D. Denslow, Abdel A. Alli

**Affiliations:** 1 Department of Physiology and Aging, College of Medicine, University of Florida, Gainesville, FL 32610, USA; 2 Department of Physiological Sciences and Center for Environmental and Human Toxicology, University of Florida, Gainesville, FL 32611, USA; 3 Department of Basic Medical Sciences, King Saud Bin Abdulaziz University for Health Sciences, Jeddah 21423, Saudi Arabia; 4 Department of Medicine, Division of Nephrology, Hypertension, and Renal Transplantation, College of Medicine, University of Florida, Gainesville, FL 32610, USA

**Keywords:** spontaneous hypertension, ENaC, extracellular vesicles, lipids

## Abstract

Hypertension remains a major problem, especially in the elderly, as it increases the risk for cardiovascular, coronary artery, cerebrovascular, and kidney diseases. Extracellular vesicles (EVs) play a role in the aging process and contribute to pathophysiology. Our goal was to examine differences in lipid profiles of urinary EVs (uEVs) collected during the inactive and active phases of aged mice and investigate whether these EVs regulate the density of lipid rafts in mouse cortical collecting duct (mpkCCD) principal cells. Here, we demonstrate the epithelial sodium channel (ENaC) inhibitor benzyl amiloride reduced systolic blood pressure in aged male mice during the inactive and active phases. Lipidomics data demonstrate differential enrichment of lipids between the two groups. For example, there are more phosphatidylethanolamine plasmalogens, particularly in the form of alkyl phosphatidylethanolamines, that are enriched in active phase uEVs compared to inactive phase uEVs from the same mice. Amiloride-sensitive transepithelial current increased more in mpkCCD cells challenged with uEVs from the active phase group. Moreover, more ENaC alpha protein was distributed to lipid raft fractions of mpkCCD cells challenged with active phase uEVs. Taken together, the identification of bioactive lipids associated with lipid rafts that are enriched in EVs released during the active phase of aged mice may offer clues to help understand lipid raft organization in recipient principal cells after EV uptake and increased renal ENaC activity, leading to a time-of-day dependent regulation of blood pressure in an aging model.

## 1. Introduction

Hypertension remains a public health problem that increases the risk of cardiovascular disease, renal disease, and cerebrovascular disease. The prevalence of hypertension significantly increases with age [1]. The kidneys are one of the most affected organs observed with normal aging. Diuretics are the first line drug for the treatment of hypertension in the elderly, but these drugs may result in exaggerated effects leading to volume depletion, hyponatremia, and hypokalemia.

Twenty-four-hour circadian rhythms regulate a myriad of physiological functions. Both central and peripheral circadian rhythms are significantly influenced by normal aging [2]. Circadian clock proteins have been demonstrated to regulate renal sodium handling [3], renal handling of water [4], and blood pressure regulation [5]. Moreover, circadian clock proteins have been demonstrated to, directly and indirectly, regulate the function of membrane proteins in the kidney including aquaporins (AQP2) [6] and epithelial sodium channel (ENaC) [5] that play an important role in total body water and electrolyte balance.

Urinary extracellular vesicles (uEVs) include exosomes [7], microvesicles [7], apoptotic bodies [7], migrasomes [8], and exomeres [9]. Virtually every cell type within the nephron releases EVs into the filtrate, and the EVs that are not taken up by downstream cells are excreted into the urine. Collectively, uEVs have been demonstrated to be enriched in important renal transport proteins including aquaporins [10,11] and epithelial sodium channels [12,13]. Recent studies have demonstrated that uEVs are a rich source of biomarkers and can be useful in the pathogenesis of hypertension [14]. Although the excretion of EVs into the urine varies on a myriad of stressors [15,16], drug treatments [17], temperature changes [18], and social habits [19], little is known about the excretion of EVs into the urine during the inactive and active phases of any animal or rodent model.

One of the objectives of this project was to investigate whether EVs are differentially released into the urine of spontaneously hypertensive aged mice during the inactive phase and active phase. A second objective was to investigate differences in the lipid profiles of EVs isolated from the urine collected during the inactive phase and active phases of these mice. The physiological relevance of the differential expression of lipids within EVs released during the inactive and active phases of aged male mice with spontaneous hypertension was investigated and discussed.

## 2. Results

### 2.1. Amiloride Reduces Systolic Blood Pressure in the Inactive and Active Cycles of Aged Male Mice with Spontaneous Hypertension

**Figure 1:**
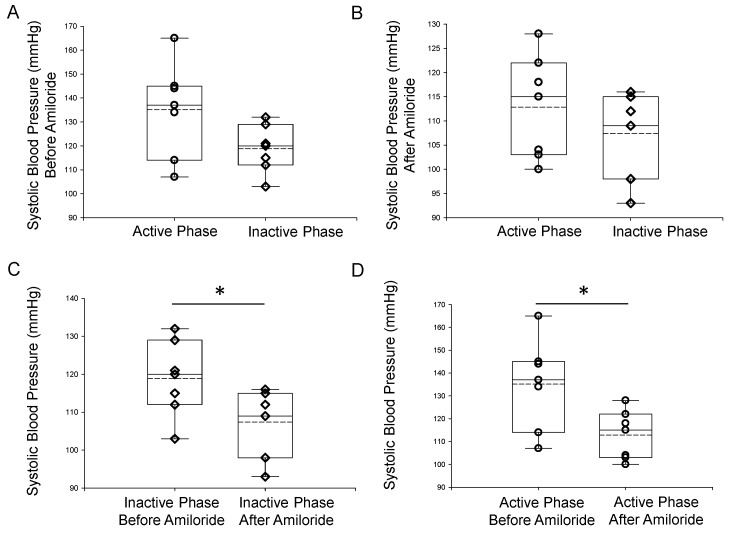
Tail cuff systolic blood pressure measurements of 18-month-old male mice during the inactive (AM) phase (IP) and active (PM) phase (AP). (**A**) Summary plot of systolic blood pressure in aged male mice taken during the inactive phase (8 AM) or during the active phase (8 PM). (**B**) Summary plot of systolic blood pressure in these mice at the same time points but after the mice were injected with benzyl amiloride. (**C**) Summary plot of systolic blood pressure during the inactive phase (8 AM) before and after the mice were injected with benzyl amiloride. (**D**) Summary plot of systolic blood pressure during the active phase (8 PM) before and after the mice were injected with benzyl amiloride. A Wilcoxon rank sum test was performed to compare the two groups. *n* = 7 mice in each group. The open circles represent individual data points from the AP group and the open triangles represent individual data points from the IP group. * Represents a *p*-value of ≤0.05.

The epithelial sodium channel plays an important role in total body salt balance and blood pressure control. Its role in the pathogenesis of salt-sensitive hypertension has been well established [20,21]. ENaC protein expression was demonstrated to be increased in the kidneys of spontaneously hypertensive rats [22]. Here, we investigated whether ENaC is associated with spontaneous hypertension in aged mice over a 24-h cycle. The ENaC inhibitor benzyl amiloride was administered and systolic blood pressure was recorded by the tail cuff method during the inactive phase (8 AM) and then again in the active phase (8 PM). Amiloride significantly reduced systolic blood pressure both during the inactive phase and active phase of aged male mice (Figure 1).

### 2.2. Urinary Electrolyte Concentration during the Inactive and Active Phases of Aged Male Mice

**Figure 2:**
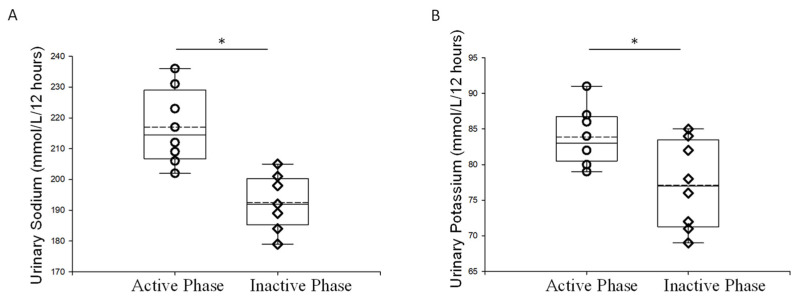
Urinary electrolyte measurements of 18-month old male mice during the inactive and active phases. (**A**) Summary plot of sodium excreted in the urine during the active and inactive phases from aged male mice (**B**) Summary plot of potassium excreted in the urine during the active and inactive phases from aged male mice. A Wilcoxon rank sum test was performed to compare the two groups. *n* = 7 in each group. The open circles represent individual data points from the AP group and the open triangles represent individual data points from the IP group. * Represents a *p*-value of ≤0.05.

Next, we examined electrolyte concentrations from urine samples collected during the inactive and active phases. Both sodium and urinary potassium levels were higher in urine samples collected during the active phase compared to the inactive phase (Figure 2).

### 2.3. Concentration of EVs Isolated from the Inactive and Active Phases of Aged Male Mice with Spontaneous Hypertension

**Figure 3:**
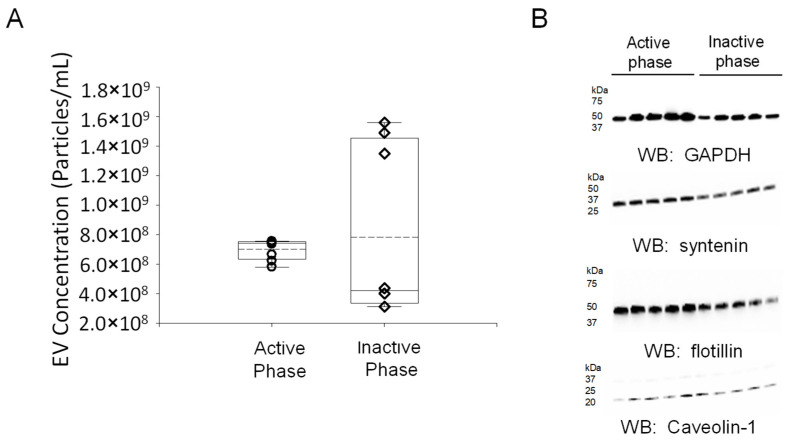
Concentration and Western blot characterization of EVs isolated from urine samples collected during the inactive and active phase of spontaneously hypertensive male C57B6 mice. (**A**) Summary plot of uEV concentration from urine produced during the active phase and collected at 8 AM and from urine produced during the inactive phase and collected at 8 PM. (**B**) Western blot (WB) analysis of multiple EV markers including GAPDH, syntenin, flotillin, and caveolin-1. A Wilcoxon rank sum test was performed to compare the two groups. *n* = 7 EV preps in each group. The open circles represent individual data points from the AP group and the open triangles represent individual data points from the IP group.

Urine contains a mixed population of EVs that originate from multiple organ systems, including the kidneys and bladder, and the molecular composition includes proteins, lipids, and metabolites [23]. Although various proteins, lipids, and metabolites have been demonstrated to be differentially expressed between the inactive and active phases in the kidneys and bladder of mice, the molecular profiles of urinary EVs isolated from urine samples collected during these phases of aged mice with spontaneous hypertension have not been investigated. Here, we performed metabolic cage studies and isolated urine during the inactive phase and active phase from male wild-type mice with spontaneous hypertension. EVs were isolated, and the concentration of each EV preparation was determined by nanoparticle tracking analysis (Figure 3).

### 2.4. Lipidomic Profiles of uEVs from Aged Male Mice with Spontaneous Hypertension

**Figure 4:**
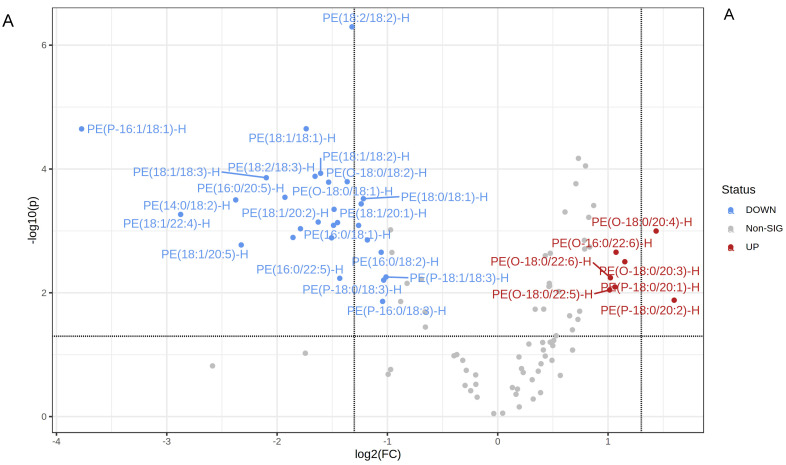

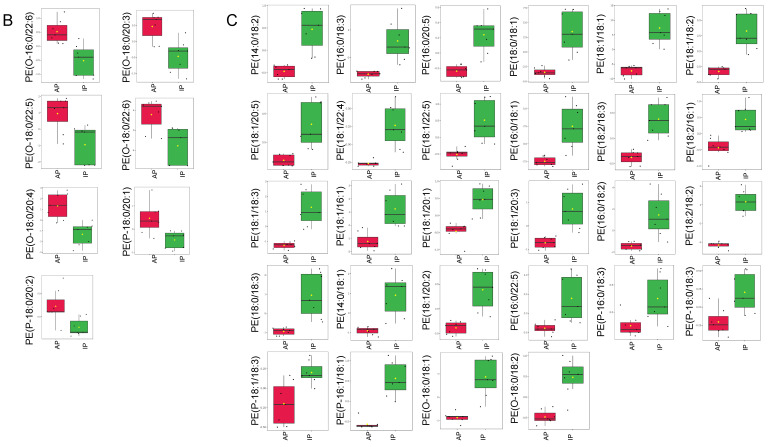
Phosphatidylethanolamine levels in uEVs isolated from urine collected during the inactive and active phases of spontaneously hypertensive aged male mice. (**A**) Volcano plot showing all PE’s increased or decreased in the EVs from the active phase (AP) group compared to the inactive phase (IP) group and all other PE’s that did not show a statistically significant difference between the two groups. The fold change threshold was set to 2.0 and the *p*-value threshold was set to 0.05. Data were normalized to the median with Pareto scaling and the plots were made in MetaboAnalyst software. (**B**) Normalized plots showing 7 PE’s that are significantly increased in EVs isolated from urine samples collected during the AP compared to the IP. (**C**) Normalized plots showing 28 PE’s that are significantly decreased in EVs from the AP group compared to the IP group. *n* = 7 samples per group. EVs isolated from AP urine are in red and EVs isolated from IP urine are in green. The concentration of each lipid on the *y*-axis is given in μM.

Our lipidomic analyses showed uEVs isolated from aged male mice are enriched in multiple types of lipids that have diverse functions. First, we examined phosphatidylethanolamines (PEs), since various modifications of these lipids have been implicated in disease mechanisms. Here, we show some PEs that are significantly increased in the EVs isolated from urine collected during the active phase compared to urine collected during the inactive phase of the same aged hypertensive mice, while the opposite trend was observed for other PEs. Additionally, there were more phosphatidylethanolamine plasmalogens overall and more alkyl-phosphatidylethanolamine (PE(O))’s in the EVs from the active phase group compared to the inactive phase group (Figure 4). The entire set of PEs are listed in the Supplemental Table S1.

**Figure 5:**
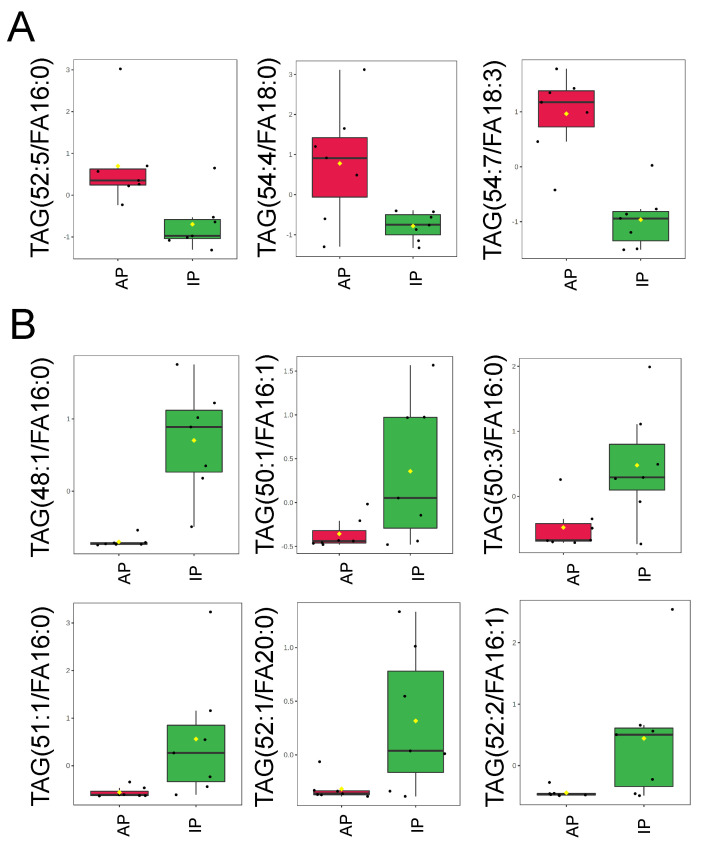
Triacylglycerol levels in uEVs isolated from urine collected during the inactive and active phases of spontaneously hypertensive aged male mice. (**A**). Normalized plots showing 3 TAGs that are significantly increased in EVs isolated from urine samples collected during the active phase (AP) compared to the inactive phase (IP). (**B**). Normalized plots showing 6 TAGs that are significantly decreased in EVs from the AP group compared to the IP group. Data were normalized to the median with Pareto scaling and the plots were made in MetaboAnalyst software. *n* = 7 samples per group. EVs isolated from AP urine are in red and EVs isolated from IP urine are in green. The concentration of each lipid on the *y*-axis is given in μM.

Since triacylglycerols are a source of energy that can regulate a myriad of mechanisms within a cell, we also compared changes in triacylglycerols (TAGs) in uEVs released during the inactive and active phases of aged male mice. Here, we demonstrate that some triacylglycerols are enriched in uEVs isolated from urine collected during the active phase compared to the inactive phase of aged male mice, while other triacylglycerols are enriched in uEVs isolated from the urine collected during the inactive phase compared to the active phase of the same mice (Figure 5). The entire set of TAGs are listed in the Supplemental Table S2.

**Figure 6:**
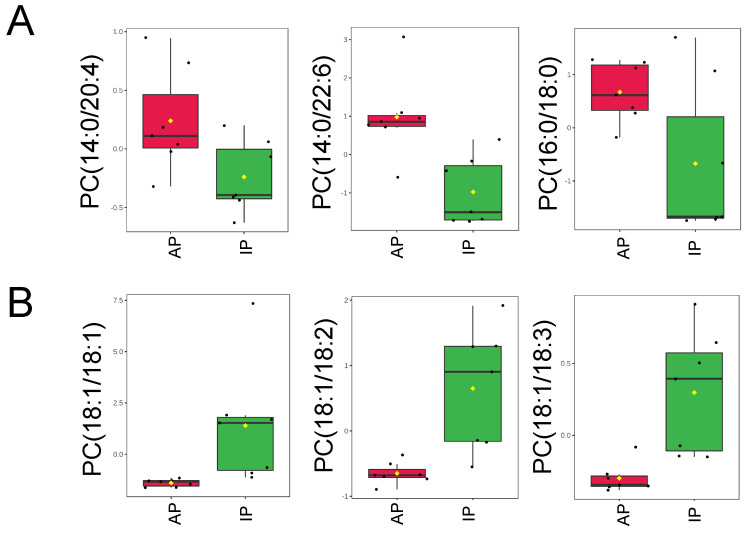
Phosphatidylcholine levels in uEVs isolated from urine collected during the inactive and active phases of spontaneously hypertensive aged male mice. (**A**) Normalized plots showing 3 PC’s that are significantly increased in EVs isolated from urine samples collected in the active phase (AP) compared to the inactive phase (IP). (**B**) Normalized plots showing 3 PC’s that are significantly decreased in EVs from the AP group compared to the IP group. Data were normalized to the median with Pareto scaling and the plots were made in MetaboAnalyst software. *n* = 7 samples per group. EVs isolated from AP urine are in red and EVs isolated from IP urine are in green. The concentration of each lipid on the y-axis is given in μM.

Next, we examined phosphatidylcholine (PC) levels in the EVs from the two groups since different forms of these lipids have been found to be involved in signal transduction. Although PCs are not known to regulate ENaC, they may regulate membrane fluidity and/or other proteins that are associated with ENaC. As shown in Figure 6A, PC(14:0/20:4), PC(14:0/22:6), and PC(16:0/18:0) were more enriched in the EVs from the active phase group compared to the inactive phase group. Conversely, PC(18:1/18:1), PC(18:1/18:2), and PC(18:1/18:3) were less concentrated in the EVs from the active phase group compared to the inactive phase group (Figure 6B). The entire set of PCs is listed in the Supplemental Table S3. Phosphatidylserines (PSs) are in Supplemental Table S4, sphingomyelins (SMs) are in Supplemental Table S5, Ceramides (CERs) are in Supplemental Table S6, phosphatidylglycerols (PGs) are in Supplemental Table S7, and monoacylglycerols (MAGs) and diacylglycerols (DAGs) are in the Supplemental Table S8.

### 2.5. uEVs from Aged Mice Augment ENaC Activity in Recipient Mouse mpkCCD Cells

**Figure 7:**
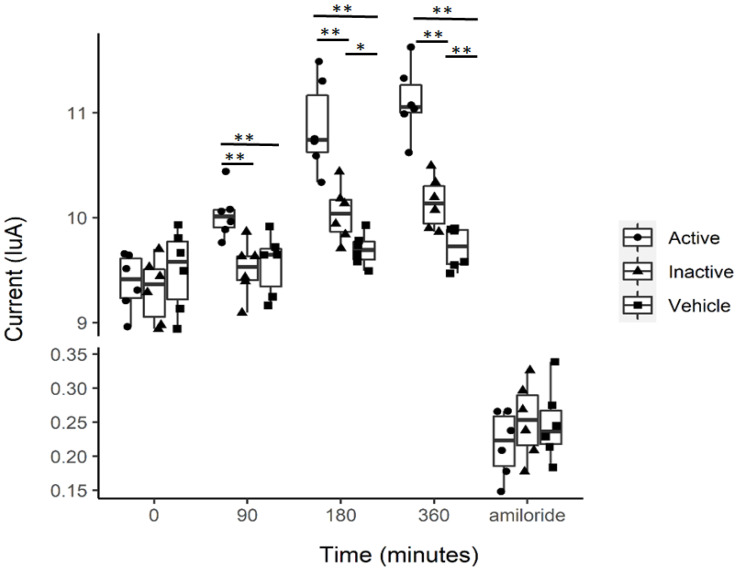
Amiloride-sensitive transepithelial current measurements in mpkCCD cells treated with 2 × 10^7^ particle/mL of EVs isolated from the urine collected during the inactive phase or active phase of spontaneously hypertensive mice. * Represents a *p*-value of ≤0.05, ** represents a *p*-value of <0.01.

Multiple studies suggest that EVs that are excreted into the urine can be taken up by recipient cells in vitro and lead to altered cellular function of membrane proteins, intercellular communication, and intracellular signaling [24,25,26,27]. Here, we investigated the effect of uEVs isolated from urine samples produced during the inactive or active phases of spontaneously hypertensive aged male mice on amiloride-sensitive transepithelial current in mouse cortical collecting duct (mpkCCD) cells. These current measurements demonstrate that compared to vehicle treatment (1× PBS), EVs isolated from urine collected during either the inactive or active phase of spontaneously hypertensive aged male mice augment amiloride-sensitive transepithelial current (Figure 7). Additionally, the amiloride-sensitive transepithelial current was greater in mpkCCD cells treated with uEVs from the active phase compared to the inactive phase (Figure 7).

### 2.6. ENaC’s Association with Lipid Rafts from mpkCCD Cells Is Augmented after Treating the Cells with EVs Isolated from the Urine Collected during the Active Phase of Aged Mice

**Figure 8:**
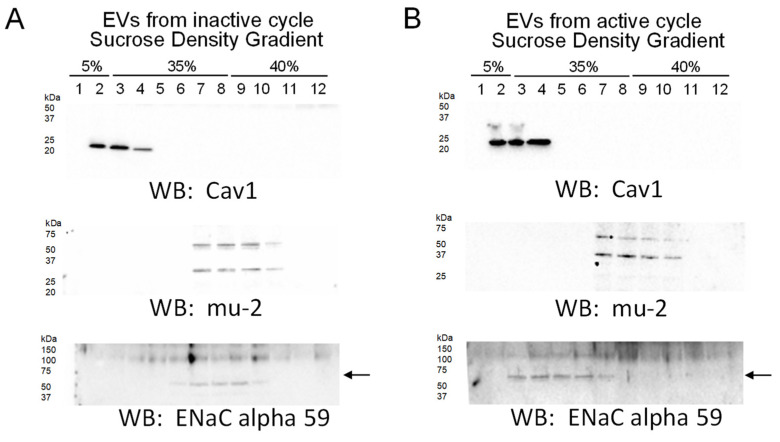
Sucrose density gradients showing the distribution of ENaC alpha subunit to lipid rafts after treating mpkCCD cells with uEVs from the active phase compared to the inactive phase. mpkCCD cells were challenged with uEVs from the inactive phase (**A**) or the active phase (**B**) before isolating lipid rafts and non-lipid rafts. Representative Western blots for ENaC alpha subunit protein expression in each fraction. Caveolin 1 (Cav1) is present in the light density gradient fractions corresponding to lipid rafts, while the mu-2 protein is present in non-lipid raft associated fractions. *n* = 2 independent experiments.

Lipid rafts are signaling platforms that have been shown to stabilize ENaC at the plasma membrane and positively regulate the activity of the channel. Our data showing lipids present in lipid rafts, including plasmalogen phosphatidylethanolamines in the form of PEPs and PEOs, are enriched more in uEVs from the active phase compared to the inactive phase of aged male mice and led us to further investigate whether these EVs can modulate the distribution of ENaC to lipid rafts in recipient collecting duct cells. We challenged mpkCCD cells with 4–6 × 10^7^ particles/mL of uEVs from either the active or inactive phases of aged male mice for 8 h before harvesting the cells for the isolation of lipid rafts. Interestingly, mpkCCD cells challenged with active phase EVs, compared to cells challenged with inactive phase EVs, showed a greater density of the lipid raft associated protein Caveolin 1 in light density sucrose gradient fractions and with more ENaC alpha protein (~60 kDa band) being distributed to these fractions (Figure 8).

## 3. Discussion

The goal of this study was to investigate how changes in lipids within small EVs less than 200 nm in diameter purified from the urine collected during the inactive or active phases of aged male mice correlate with blood pressure regulation over a 24-h cycle. The rationale for focusing on smaller EVs is because these vesicles encompass exosomes, which have been previously demonstrated to allow for intercellular communication within the nephron and they have been demonstrated to regulate ENaC activity [26].

Aging is associated with an increase in reactive oxygen species that can damage cellular proteins and lipids. Eum et al. performed a comprehensive lipidomic study on young and old mice and investigated various targets including the kidneys [28]. Braun et al. investigated mechanisms associated with kidney aging and identified age-related differences in phosphatidylethanolamines, sphingomyelins, ceramides, phosphatidylserines, and phosphatidylcholines [29]. To the best of our knowledge, our study is the first to compare lipid profiles of uEVs released during the inactive and active phases of aged male mice with spontaneous hypertension.

Braun et al. demonstrated an overall decrease in the total content of PS, PC, PE, SM in aged compared to young kidneys [29]. Dorrance et al. reported a higher concentration of sphingomyelin in the membranes of hypertensive rats compared to control animals [30]. Noh et al. demonstrated that the kidneys of older mice have lower levels of sphingomyelins and phosphatidylcholines compared to younger mice [31]. Our study investigates the lipid profiles of EVs isolated from the urine collected during the inactive phase and active phase of aged male mice with spontaneous hypertension. Our lipidomic data demonstrate that some PEs, TAGs, and PCs are enriched in EVs purified from the urine produced during the inactive phase, while other PEs, TAGs, and PCs are enriched in EVs from the active phase urine of spontaneously hypertensive aged male mice.

In our blood pressure studies, the ENaC inhibitor amiloride significantly reduced blood pressure in spontaneously hypertensive aged mice. Moreover, we demonstrated that the blood pressure is higher in these aged mice during the animals’ active phase compared to their inactive phase. Since these data implicate a role for elevated ENaC activity in a time-of-day dependent manner, we investigated differential enrichment of lipids in uEVs during the animals’ inactive and active phases. uEVs are a mixed population of EVs that originate mainly from different cell types along the nephron. Published studies have demonstrated that ENaC is positively regulated by various lipids, [32,33,34] and the channel is present in lipid rafts in Xenopus A6 cells [35] and mouse mpkCCD [36,37]. Since plasmalogen phosphatidylethanolamines, PEPs and PEOs, are considered bioactive lipids and have been demonstrated to be associated with lipid rafts, we further investigated whether inactive phase EVs and active phase EVs can differentially modulate the distribution of ENaC to lipid rafts in recipient mpkCCD cells. Intriguingly, mpkCCD cells challenged with active phase EVs enriched more in plasmalogen PE’s, in particular PEO’s, compared to inactive phase EVs, resulted in more ENaC alpha protein being distributed to light density gradient fractions associated with lipid rafts (Figure 8). Presumably, the plasmalogen PEs become distributed into the apical plasma membranes of the recipient mpkCCD cells after they take up the EVs, resulting in an increase in the local concentration of lipid rafts in close proximity to ENaC. This, in turn, would result in the stabilization of ENaC at the plasma membrane and an increase in overall channel activity, which is consistent with our amiloride-sensitive transepithelial current experiments that demonstrated higher current in ENaC expressing mpkCCD cells challenged with active phase EVs compared to inactive phase EVs, or the vehicle alone (Figure 7). The physiological significance of more renal ENaC being associated with lipid rafts is an increase in overall sodium retention in the body and a subsequent increase in systemic blood pressure. Furthermore, biochemical data on the unique lipid profiles of EVs released during the inactive and active phases of aged mice (Figure 4), along with cellular physiology data on EV uptake by mpkCCD cells (Figure 8), suggest a novel mechanism by which ENaC can be regulated in a time-of-day dependent manner.

One limitation of our study is that we were not able to compare relative amounts of lipids in uEVs from the kidneys of young and old spontaneously hypertensive mice since young healthy mice do not typically develop spontaneous hypertension. Another limitation of our study is that we did not investigate sex differences in bioactive lipids directly in kidney tissue of aged mice, in a time-of-day dependent manner, since aged female mice generally have lower blood pressure compared to male mice, and we euthanized all of our mice at the same time of day. A third limitation of our study is that our lipidomic analysis was focused on uEVs less than 200μm in diameter because we were more interested in the role of exosomes in regulating ENaC-dependent blood pressure, although we realize other types of EVs may be involved, and a comparative lipidomic analysis of the different types of uEVs may be interesting. Finally, although the majority of uEVs are thought to originate from the polarized epithelial cells of the kidney, it is difficult to sort uEVs, and we were not able to determine the relative amounts of EVs released by the various cell types in the nephron.

Taken together, the data presented in this study suggest that the packaging of EV lipids is differentially regulated over a 24-h cycle. The data also suggest EVs excreted into the urine during the active phases of spontaneously hypertensive male mice can regulate the density of lipid rafts and the recruitment of endogenous ENaC protein. These data warrant the need for additional experiments aimed at investigating membrane dynamics and the rate of recycling of ENaC to and from the membrane in a model of spontaneous hypertension.

## 4. Materials and Methods

### 4.1. Animal Diet and Metabolic Cage Studies

C57Bl6 male mice were purchased from the Jackson Laboratory (Bar Harbor, ME, USA) and aged to 18 months. The mice were subject to metabolic cage studies for a five-week period. They were kept on a 12-h light/12-h day cycle and given ad libitum access to food (Envigo, Indianapolis, IN, USA) and water. Water intake and urine output were measured twice daily every 12 h. All animal studies were performed under an approved Institutional Animal Care and Use Committee protocol at the University of Florida.

### 4.2. Benzyl Amiloride Treatment

Intraperitoneal injections of 2 mg/kg Benzamil (Benzyl Amiloride) (Sigma, St. Louis, MO, USA) were performed for 3 consecutive days.

### 4.3. Blood Pressure Measurements

A total of seven C57Bl6 male mice 18 months of age were subject to blood pressure measurements by the tail-cuff method. Blood pressure measurements were performed twice a day on separate days with a 12-h difference (8 AM and 8 PM) at the beginning and end of the study, and before and after amiloride injections. Mice were euthanized at the end of the study by cervical dislocation.

### 4.4. Electrolyte Measurements

Urinary sodium and potassium levels were measured using an electrolyte analyzer (Diamond Diagnostics; Holliston, MA, USA). Briefly, each urine sample was centrifuged at 13,000× *g* for six minutes, aliquoted, and then diluted (1:14) with urine diluent (Diamond Diagnostics). The samples were then analyzed in urine mode.

### 4.5. Isolation and Characterization of Urinary Extracellular Vesicles

Ten milliliters of pooled urine samples (from the same mouse over the course of 1 week) collected at 8 PM (representing urine produced during the inactive phase) or urine samples collected at 8 AM (representing urine produced during the active phase) were cleared by centrifugation for 10 min at 1000× *g*. The supernatants were filtered through Nalgene 0.2 μm filters, and the supernatants were then subject to ultracentrifugation at 118,000× *g* for 70 min. The EV pellets were resuspended in ultrapure 1× PBS and then subjected to ultracentrifugation again at the same speed and duration. The isolated EVs that were reconstituted in 0.2 μm filtered ultra-pure 1× PBS were stored at minus 80 °C in small aliquots, as previously reported [38]. The concentration and size of the uEVs were determined by nanoparticle tracking analysis using an NS300 machine (Malvern Panalytical, Malvern, UK). Fifty microliter aliquots of the uEVs were lysed by sonication in Laemmli sample buffer (BioRad; Hercules, CA, USA) to probe for EV markers by Western blotting.

### 4.6. Western Blotting of Lysed uEVs

A BCA Assay (Thermo Fisher Scientific; Waltham, MA, USA) was performed to determine the protein concentration of lysed uEVs samples. Fifty micrograms of total protein were resolved on 4–20% Criterion precast gels (Thermo Fisher Scientific) at 200 V for one hour on a Criterion electrophoresis system (BioRad). The proteins were electrically transferred to nitrocellulose membranes (ThermoFisher Scientific) at room temperature using a Criterion transfer apparatus (BioRad), blocked in nonfat dry milk in 1× TBS (BioRad) for one hour, and incubated overnight with a 1:1000 dilution of Syntenin (ab19903; Abcam, Waltham, MA), GAPDH (Cell Signaling Tech (Danvers, MA, USA), 3683), Flotillin-2 (3244; Cell Signaling), or caveolin-1 (ab2910, Abcam) antibody. The membranes were then washed with 1× TBS (BioRad) and incubated with goat anti-rabbit secondary antibody (BioRad) at a 1:3000 dilution for 1 h at room temperature. After another round of washes, ECL Select (BioRad) was incubated with the membranes for 7 min. The blots were then developed using a Bio-Rad imager.

### 4.7. Extraction of Lipids

Lipids were extracted from an equal number of EVs (6.29 × 10^9^) per sample according to the Bligh and Dyer method [39]. Briefly, the solution containing EVs was adjusted to 1 mL using water in a 10-mL glass vial. After incubation on ice for 10 min, 2 mL methanol, and 0.9 mL methylene chloride were added and the solution was vortexed for 30 s. EquiSPLASH Lipidomix (Avanti Polar Lipids, Inc., Alabaster, AL, USA), a mixture of 13 deuterated lipids was used as an internal standard. The composition of the internal standard mixture was described in our previous publication [14]. The internal standard was diluted 5 times using cold methanol to adjust each deuterated lipid’s concentration to 20 µg·mL^−1^, and then 50 µL was spiked into each sample. Samples were incubated at room temperature for 30 min, followed by adding 1 mL water and 0.9 mL methylene chloride. The vials were inverted 10 times and centrifuged at 200× *g* for 10 min. The organic lower phase was carefully collected using a glass Pasteur pipette. To improve the recovery of lipids, the centrifugation was repeated by adding 2 mL methylene chloride and centrifugation. The lower phase was collected, added to the first extract, and dried using a gentle stream of N_2_. Lipids were resuspended in 50 µL 96% ethanol and analyzed using liquid chromatography with tandem mass spectrometry (LC-MS/MS). Lipids were extracted from uEVs of seven different male mice 18 months of age with a systolic blood pressure of 134 or greater.

### 4.8. Liquid Chromatography with Tandem Mass Spectrometry

Five µL of the extracted lipids were injected into ultra-high-performance liquid chromatography (UHPLC, Shimadzu Co., Kyoto, Japan) coupled to a QTRAP 6500 mass spectrometer (AB SCIEX, Redwood Shores, CA, USA). Lipids were separated using a binary gradient of acetonitrile: water 95:5 (vol/vol) for mobile phase A and 50:50 (vol/vol) for mobile phase B in an XBridge Amide 3.5 μm, 4.6 × 150 mm column (Waters, Dublin, Ireland). Both mobile phases contained 1 mM ammonium acetate, and the pH of the solutions was adjusted to 8.2. A linear gradient of solvent B increased to 6% in 6 min, to 25% within 4 min, to 98% within 1 min, and then to 100% within 2 min with a flow rate of 0.7 mL.min^−1^. In order to avoid carryover, the flow rate was increased to 1.5 mL.min^−1^ for 3 min at the end of each run to flush the column and tubing. The needle was washed using 500 μL isopropanol, and blanks were run in sample intervals.

A scheduled Multiple Reaction Monitoring algorithm was applied to detect lipids in both negative and positive ion modes. The declustering potentials of the electrospray ionization source for positive and negative modes were set to 60 and 80, respectively. The entrance and collision cell exit potentials were adjusted to 10 and 15, respectively. The ion spray voltage was set at 4.5 kV with a temperature of 300 °C. Collision energy varied from 25 to 60 depending on the lipid species. Each sample was injected twice as technical replicates.

### 4.9. Quantification of Lipids

Over 1100 lipid species were scanned from 19 lipid groups including phosphatidylcholine (PC), phosphatidylethanolamine (PE), phosphatidylinositol (PI), phosphatidylglycerol (PG), phosphatidylserine (PS), lysophosphatidylcholine (LPC), lysophosphatidylethanolamine (LPE), lysophosphatidylinositol (LPI), lysophosphatidylglycerol (LPG), lysophosphatidylserine (LPS), triacylglycerol (TAG), diacylglycerol (DAG), monoacylglycerol (MAG), cholesterol ester (CE), sphingomyelin (SM), ceramide (CER), hexosylceramide (HCER), dihydroceramide (DCER), and lactosylceramide (LCER) using Analyst software (ver. 1.6.2). Extraction and ionization efficiencies were corrected for each lipid class using the appropriate internal standard for the class. Relative concentrations of lipids were calculated based on the peak area of the analytes to the peak area of the surrogate internal standard using MultiQuant software (ver. 3.0.3). The relative quantity of lipids was calculated based on the concentration of the surrogate internal standard for each class.

### 4.10. mpkCCD Cell Culture

mpkCCD principal cells were plated on 6-well permeable supports (Corning Inc., Corning, NY, USA), and the cells were maintained in complete growth media containing 20 mM HEPES, 50 nM dexamethasone, 1 nM triiodothyronine, 2 mM l-glutamine, 0.1% penicillin-streptomycin, and 2% heat-inactivated FBS. The media was replaced every other day. The cells were used for experiments 10 days after reaching confluence.

### 4.11. Amiloride-Sensitive Transepithelial Current Measurements

mpkCCD cells were cultured on 12-well permeable supports. The cells were subject to transepithelial voltage and resistance measurements using an epithelial volt/ohm meter (EVOM2) (World Precision Instruments; Sarasota, FL, USA) 5 days after the cells reached confluence. At the end of the experiment, the cells were treated with 1 µM amiloride (Sigma) and current was calculated by Ohm’s law.

### 4.12. Sucrose Density Gradient Assays

Lipid rafts and non-lipid raft associated fractions were isolated from mpkCCD cells, as previously described by our group [37].

### 4.13. Statistical Analyses

A Student paired *t*-test was used to make comparisons between the inactive and active cycles of each group. Two-way repeated measures analysis was used to analyze current measurements accounting for correlations among repeated measurements at each phase (active or inactive) or vehicle control and time points (0 min, 90 min, 180 min, 360 min, after amiloride injection). Bonferroni adjustments were used for multiple comparisons at each timepoint. For the blood pressure and electrolyte data, SigmaPlot 14.0 (Systat Software, Inc., Point Richmond, CA, USA) was used for the analysis and plots. MetaboAnalyst 5.0 software (https://www.metaboanalyst.ca) was used for the analysis and plots of all the lipidomic data. The SAS 9.4 (v.9.4, Cary, NC, USA) and R 4.2.1 software were used to perform the indicated analyses and plot the raw electrophysiology data.

## Data Availability

The individual data points are shown within the plots and the entire lipidomic data set is shown in the supplementary tables.

## Supplementary Materials

The following supporting information can be downloaded at: https://www.mdpi.com/article/10.3390/ijms232315397/s1.
