# Supplementary material for: Lipid Profiles of Urinary Extracellular Vesicles Released during the Inactive and Active Phases of Aged Male Mice with Spontaneous Hypertension
Source: Int J Mol Sci. 2022 Dec 6;23(23):15397. doi: 10.3390/ijms232315397 (PMC9739303; doi:10.3390/ijms232315397)
Supplement: Supplementary file 1 [file ijms-23-15397-s001.zip › ijms-1963408-supplementary.pdf]

***Supplemental Table S1**:* PE, PE(O), PE(P) concentration (in μM) in EVs isolated from urine collected during the active phase (AP) compared to the inactive phase (IP)

Lipid	AP1	AP2	AP3	AP4	AP5	AP6	AP7	IP1	IP2	IP3	IP4	IP5	IP6	IP7
PE(14:0/18:1)-H	0.002516305	0.0036448	0.002357027	0.006633433	0.003792544	0.006653325	0.001610347	0.039250389	0.026059017	0.011860772	0.008901035	0.008181049	0.010958124	0.016661407
PE(14:0/18:2)-H	0.000681301	0.00009	0.000321352	0.001288856	0.00009	0.001130178	0.000253657	0.007867316	0.006139733	0.002470851	0.002109229	0.001935869	0.002194662	0.003049408
PE(16:0/16:0)-H	0.005607958	0.00545312	0.00612336	0.008159968	0.004302998	0.007338828	0.002583632	0.019724695	0.016035262	0.00945503	0.005037452	0.005597208	0.006688572	0.012387139
PE(16:0/18:1)-H	0.037161891	0.04171884	0.038758944	0.095642984	0.037416561	0.093884177	0.01856061	0.362924068	0.281027176	0.129150735	0.087284147	0.079019916	0.07184504	0.193416136
PE(16:0/18:2)-H	0.01530694	0.016509582	0.016232556	0.037064035	0.016284871	0.037586215	0.007822212	0.139833453	0.102134774	0.04983548	0.033129701	0.027287037	0.028018574	0.073703758
PE(16:0/18:3)-H	0.001216915	0.001176825	0.001008317	0.002821143	0.001397271	0.002801961	0.000398405	0.018735671	0.011428003	0.005328807	0.004002834	0.003268313	0.003387813	0.007748153
PE(16:0/20:1)-H	0.000577842	0.00053648	0.000740332	0.000923691	0.000544905	0.001284918	0.000511655	0.002507823	0.002201062	0.001251139	0.000600936	0.00074041	0.000762674	0.001857269
PE(16:0/20:2)-H	0.000911593	0.000565592	0.000799226	0.001263047	0.00033982	0.001459068	0.000236036	0.002728018	0.002194934	0.001464961	0.000686947	0.00074164	0.000689246	0.001964492
PE(16:0/20:3)-H	0.002756985	0.001668457	0.002605172	0.00562491	0.002068787	0.005282943	0.001039434	0.0113475	0.008984854	0.005353007	0.003049743	0.002667995	0.002449373	0.005684539
PE(16:0/20:4)-H	0.010746998	0.007533177	0.010373483	0.026232905	0.00804022	0.025304926	0.004142462	0.029858375	0.02538352	0.015666387	0.007983182	0.007412267	0.007587873	0.021728187
PE(16:0/20:5)-H	0.00009	0.00009	0.00009	0.000463874	0.000179945	0.000468529	0.00009	0.001895551	0.00172587	0.00070998	0.000649145	0.000495708	0.000641848	0.001150286
PE(16:0/22:4)-H	0.000379229	0.000419409	0.000392117	0.000818457	0.000501879	0.001040209	0.00009	0.002127048	0.001704213	0.000963323	0.000501158	0.000710518	0.000523227	0.001355312
PE(16:0/22:5)-H	0.000799332	0.000750158	0.000746924	0.002340045	0.00041198	0.001747338	0.000531051	0.008785145	0.005826675	0.003048742	0.002213483	0.002505854	0.001408474	0.003337538
PE(16:0/22:6)-H	0.004526347	0.003127918	0.003941975	0.009709797	0.003003297	0.008811944	0.001722454	0.008787989	0.008183162	0.006038581	0.002392559	0.002558326	0.00246574	0.007661066
PE(18:0/14:0)-H	0.000843692	0.000405754	0.000788985	0.000995191	0.000403011	0.001047177	0.000190327	0.004034791	0.003451468	0.001503156	0.000916272	0.001031105	0.005637059	0.002394829
PE(18:0/16:0)-H	0.001501992	0.001983213	0.001321664	0.00338675	0.001741976	0.003433727	0.000785424	0.012505153	0.010456406	0.00517148	0.002998396	0.002367802	0.002532754	0.006688876

PE(18:0/16:1)-H	0.00080452	0.000902493	0.000697551	0.002171261	0.0008179	0.002378712	0.000277112	0.008451393	0.006647929	0.00495323	0.001935833	0.00168469	0.021202909	0.006096839
PE(18:0/18:0)-H	0.003020819	0.002444135	0.00310343	0.005277542	0.003294569	0.005603792	0.001665328	0.022488797	0.019717178	0.010402992	0.004975649	0.004131209	0.004072483	0.012929108
PE(18:0/18:1)-H	0.071064035	0.064635327	0.065265836	0.164177674	0.068978351	0.154468063	0.032342602	0.569200476	0.498484026	0.258096645	0.152386471	0.142165874	0.13361758	0.356960003
PE(18:0/18:2)-H	0.057927229	0.043600959	0.054674303	0.111609729	0.043377954	0.109180028	0.023441818	0.293991021	0.235405234	0.13230249	0.074643404	0.070706095	0.066496132	0.183214447
PE(18:0/18:3)-H	0.003171453	0.004281641	0.003582392	0.009481987	0.004042509	0.009015516	0.001986101	0.044953938	0.032893694	0.015676233	0.011490127	0.010733053	0.00858498	0.01985434
PE(18:0/20:2)-H	0.001681385	0.000793958	0.001201876	0.001915069	0.00064408	0.002447032	0.000297175	0.004808037	0.003897616	0.002052627	0.000567967	0.001289463	0.000920083	0.003581514
PE(18:0/20:3)-H	0.00992866	0.006730318	0.007737085	0.022053409	0.007003746	0.019801051	0.002632359	0.034517308	0.026337023	0.012560508	0.008905089	0.005380369	0.007062394	0.015756573
PE(18:0/20:4)-H	0.056112937	0.037449849	0.056969003	0.109153087	0.03881353	0.107736064	0.022236126	0.106446078	0.107383718	0.0758054	0.029291586	0.026258297	0.034224802	0.102345138
PE(18:0/20:5)-H	0.000934839	0.000912971	0.000980328	0.002369444	0.001032263	0.001854295	0.000526144	0.006262602	0.004745296	0.002216591	0.001915771	0.001899639	0.001531664	0.003464683
PE(18:0/22:4)-H	0.000934892	0.001049476	0.001237062	0.002647221	0.00042616	0.002683971	0.000440119	0.004484021	0.003217674	0.001837541	0.001190948	0.000887623	0.000821511	0.002756721
PE(18:0/22:5)-H	0.002063971	0.002478172	0.002133604	0.004403539	0.002292238	0.00478923	0.001042929	0.013264372	0.009746167	0.005145107	0.003226219	0.003322464	0.002948196	0.006782274
PE(18:0/22:6)-H	0.01193905	0.007319774	0.010937872	0.019099633	0.006720053	0.019167357	0.005169836	0.017285156	0.016430716	0.013000736	0.004502631	0.003695261	0.005435745	0.016683619
PE(18:1/16:1)-H	0.004695936	0.005754236	0.003867762	0.020885462	0.005905377	0.02342001	0.002592127	0.03871639	0.070844552	0.069177295	0.009048051	0.008907128	0.016440102	0.088293569
PE(18:1/18:1)-H	0.124553317	0.196853168	0.108271076	0.370824713	0.206306152	0.374453248	0.071916839	1.926148433	1.616136316	0.945933804	0.497701357	0.464239912	0.467945349	1.268474323
PE(18:1/18:2)-H	0.034073417	0.041619239	0.028613105	0.088785113	0.046833984	0.089438939	0.016920804	0.436462019	0.333211859	0.190762676	0.10786668	0.107848536	0.098851642	0.260552274
PE(18:1/18:3)-H	0.002702194	0.004593756	0.002452191	0.00753379	0.004972156	0.007771091	0.001790679	0.062049209	0.045250061	0.023376596	0.01531444	0.01411527	0.013046572	0.02988278
PE(18:1/20:1)-H	0.00136799	0.001593988	0.0013132	0.002842795	0.001307001	0.002897408	0.00009	0.009977137	0.009151854	0.004933499	0.002573905	0.002342097	0.002806089	0.00595368

PE(18:1/20:2)-H	0.000537947	0.000729517	0.000522502	0.001621901	0.000695347	0.001680316	0.000201244	0.006565997	0.004825129	0.002624325	0.001629579	0.001699994	0.001824154	0.003438741
PE(18:1/20:3)-H	0.00201115	0.002807111	0.002105851	0.006283132	0.002360757	0.005944502	0.0007655	0.025773727	0.020048635	0.009671332	0.005781236	0.006328809	0.005063094	0.012999032
PE(18:1/20:4)-H	0.011285837	0.009276439	0.009561456	0.031191489	0.00928232	0.030149844	0.005056789	0.04517263	0.038087751	0.026413286	0.011202567	0.010027843	0.011586484	0.032372121
PE(18:1/20:5)-H	0.00009	0.00030453	0.00009	0.000704829	0.00009	0.000682345	0.00009	0.00419698	0.00244937	0.001269236	0.001101674	0.001181663	0.000783962	0.001433881
PE(18:1/22:4)-H	0.00009	0.00009	0.00009	0.00009	0.00009	0.00009	0.00009	0.002843186	0.001847354	0.00066301	0.000645043	0.000610915	0.000526091	0.000905842
PE(18:1/22:5)-H	0.00009	0.000990808	0.000639516	0.001828153	0.001316476	0.001786701	0.000516605	0.012039147	0.0084538	0.004007495	0.002933434	0.003193473	0.002276782	0.004860619
PE(18:1/22:6)-H	0.002629821	0.002361424	0.002610329	0.006750485	0.002893874	0.006881234	0.001253598	0.007124861	0.006938336	0.005745782	0.0018264	0.002028548	0.002217511	0.009728197
PE(18:2/16:1)-H	0.00009	0.00116119	0.001178924	0.003131135	0.001086496	0.003420297	0.000449559	0.005235369	0.010109104	0.008143489	0.001443333	0.001459649	0.002155459	0.011791437
PE(18:2/18:2)-H	0.005740574	0.006576299	0.005672519	0.012222262	0.005896138	0.01230367	0.003342846	0.046059983	0.048025952	0.031891473	0.013335564	0.011355521	0.012741866	0.041240282
PE(18:2/18:3)-H	0.00024862	0.000604173	0.000294856	0.000950196	0.000603064	0.001018278	0.000382072	0.005818419	0.004874097	0.002575695	0.001408825	0.001388766	0.001212985	0.00339479
PE(18:2/20:1)-H	0.000509097	0.000330233	0.000380545	0.000782543	0.00009	0.000822785	0.00009	0.001959424	0.001362953	0.001304708	0.000567859	0.000386509	0.000525329	0.001348573
PE(18:2/20:3)-H	0.00009	0.00009	0.00009	0.000396792	0.000513831	0.00066005	0.00009	0.002732747	0.001834455	0.00085469	0.000587949	0.00009	0.00009	0.00009
PE(18:2/20:4)-H	0.000807008	0.00009	0.00009	0.00009	0.00009	0.001635463	0.00009	0.003358229	0.002071576	0.001405864	0.00009	0.00009	0.000767774	0.001947653
PE(O-16:0/18:3)-H	0.000609686	0.001065992	0.000710035	0.000861055	0.000666043	0.000646079	0.000830577	0.000972363	0.000751045	0.001068416	0.000982454	0.000826394	0.000845377	0.000737055

PE(O-16:0/20:4)-H	0.009147325	0.007719488	0.006987306	0.005166675	0.007193675	0.007577148	0.008599055	0.007966056	0.008362551	0.008439905	0.008270186	0.007396889	0.008716011	0.008010892
PE(O-16:0/20:5)-H	0.003325163	0.003667397	0.003399508	0.003776773	0.003885687	0.003018615	0.003699709	0.003647026	0.002930855	0.004190862	0.00371733	0.003493679	0.00314219	0.004315507
PE(O-16:0/22:4)-H	0.000820218	0.000665809	0.000858943	0.000886764	0.00009	0.001120952	0.00009	0.000644594	0.001174622	0.001378267	0.00009	0.00009	0.000387882	0.001811734
PE(O-16:0/22:5)-H	0.002262794	0.001208459	0.002371807	0.001810226	0.001685916	0.001854434	0.001009428	0.000962245	0.003354025	0.004558342	0.00009	0.000391297	0.001328105	0.005210648
PE(O-16:0/22:6)-H	0.004756753	0.004925091	0.004526589	0.006688032	0.003975809	0.006646515	0.003118258	0.002429563	0.008513324	0.008428312	0.000449429	0.000650986	0.002264166	0.011830248
PE(O-18:0/18:0)-H	0.001421367	0.00119667	0.001703768	0.001671239	0.001695845	0.001869356	0.0007456	0.001419391	0.002570677	0.002266376	0.000445356	0.000429525	0.000987215	0.002491547
PE(O-18:0/18:1)-H	0.001972886	0.003032561	0.004847729	0.006113531	0.002694462	0.006386068	0.001445049	0.030049022	0.02661902	0.016218434	0.008338894	0.00776785	0.007958036	0.014121554
PE(O-18:0/18:2)-H	0.001606851	0.001386907	0.000828788	0.001781795	0.000926145	0.002135629	0.000534207	0.009406034	0.007376129	0.008064003	0.002008444	0.002654003	0.002843216	0.004288772
PE(O-18:0/20:3)-H	0.004038652	0.004120938	0.004715855	0.004202209	0.003916888	0.00425405	0.001874012	0.002806383	0.006112372	0.006043117	0.000389028	0.00009	0.00182557	0.009467271
PE(O-18:0/20:4)-H	0.023828367	0.026736498	0.033513377	0.027216506	0.029544867	0.029808163	0.010128749	0.012790896	0.037870071	0.033183797	0.002146344	0.00009	0.010346247	0.048056549
PE(O-18:0/22:5)-H	0.021091764	0.017606793	0.023889376	0.021511406	0.017949685	0.016700367	0.01112611	0.007232594	0.038487461	0.036292931	0.001974407	0.001995622	0.012228201	0.036646464
PE(O-18:0/22:6)-H	0.220743757	0.174586347	0.236145109	0.206321055	0.200632079	0.218527556	0.104477575	0.079320104	0.348127094	0.34330984	0.021807393	0.021148412	0.131486256	0.419532445

PE(P-16:0/18:0)-H	0.001781903	0.00009	0.001536101	0.002756152	0.000776627	0.002238848	0.000483171	0.002936952	0.002333924	0.002065567	0.000932939	0.000782591	0.001015759	0.0034266
PE(P-16:0/18:1)-H	0.060709486	0.026738699	0.055355147	0.099450012	0.027027398	0.098578365	0.020110179	0.10054455	0.113142141	0.088423305	0.028267896	0.026835125	0.032861549	0.120001363
PE(P-16:0/18:2)-H	0.010262582	0.006622187	0.009709253	0.020750427	0.005962217	0.020751638	0.003388443	0.031962023	0.029402524	0.020288002	0.008117759	0.007003726	0.00895761	0.025953103
PE(P-16:0/18:3)-H	0.000511438	0.000575643	0.000410729	0.001373379	0.000416714	0.00084969	0.000554938	0.004842895	0.00321899	0.001492252	0.001027249	0.001322092	0.000868567	0.002461697
PE(P-16:0/20:1)-H	0.005771596	0.002812781	0.00483866	0.005914492	0.002780223	0.005156721	0.001588462	0.003134423	0.007660151	0.008683825	0.000674803	0.000792271	0.002525287	0.00952921
PE(P-16:0/20:2)-H	0.003726972	0.001220397	0.003763189	0.004726306	0.001467859	0.004987682	0.001248698	0.003045264	0.005646029	0.004400983	0.001131376	0.000754067	0.001491029	0.006261162
PE(P-16:0/20:3)-H	0.00935419	0.002933572	0.01004373	0.020841398	0.003211384	0.020368305	0.003762827	0.01717497	0.018699622	0.013943647	0.004587301	0.003856917	0.005969304	0.020115628
PE(P-16:0/20:4)-H	0.048796758	0.029400695	0.045028521	0.136673355	0.027103858	0.141060834	0.01857583	0.112300568	0.107254353	0.070240924	0.03078821	0.028007101	0.032901583	0.095322899
PE(P-16:0/20:5)-H	0.000740753	0.000320078	0.000960314	0.001642012	0.000388263	0.002153382	0.000268117	0.00270786	0.002005443	0.001392454	0.000843557	0.00063949	0.000677739	0.002005779
PE(P-16:0/22:4)-H	0.005664755	0.003457092	0.006944441	0.010425149	0.002958287	0.010604567	0.002465772	0.006689485	0.010159147	0.008187209	0.00156922	0.001345757	0.002657019	0.010204498
PE(P-16:0/22:5)-H	0.008782963	0.006329205	0.007705032	0.011391243	0.005756801	0.011434786	0.003592812	0.007859548	0.018432392	0.016601501	0.002506215	0.002149019	0.005088732	0.024665422
PE(P-16:0/22:6)-H	0.025147692	0.014079898	0.024332134	0.031307521	0.015673472	0.031562878	0.009773702	0.019273829	0.046580708	0.050506649	0.005095812	0.004387621	0.013989458	0.063155215

PE(P-16:1/18:1)-H	0.00009	0.00070789	0.00009	0.00009	0.00009	0.00009	0.00009	0.008188725	0.007176434	0.004159602	0.002302814	0.002529974	0.002106078	0.004008298
PE(P-18:0/16:1)-H	0.002628682	0.001552158	0.002880882	0.002756105	0.000520918	0.002258168	0.000980664	0.002777453	0.003454303	0.002757126	0.000645069	0.00077313	0.001041666	0.003106558
PE(P-18:0/18:1)-H	0.023404969	0.012746648	0.022863869	0.028517683	0.012187033	0.030229788	0.007868003	0.034098054	0.036583144	0.02698819	0.007474834	0.008982636	0.011381946	0.037852746
PE(P-18:0/18:2)-H	0.015509284	0.00892771	0.015541931	0.023145536	0.008595016	0.022030897	0.006146999	0.021001458	0.031098888	0.022451679	0.006216365	0.006246174	0.009158441	0.034392865
PE(P-18:0/18:3)-H	0.000273392	0.000645178	0.000429416	0.000636726	0.000246165	0.000995658	0.00009	0.00282211	0.002149835	0.001259835	0.000744357	0.000649089	0.000613166	0.001400205
PE(P-18:0/20:1)-H	0.006897275	0.003543871	0.005436167	0.003957986	0.003438512	0.005089643	0.00240349	0.002955756	0.007526645	0.007250215	0.000595429	0.000839964	0.002207188	0.008547146
PE(P-18:0/20:2)-H	0.001464142	0.000657631	0.001302321	0.001164846	0.000592028	0.001173941	0.00009	0.000733849	0.001750817	0.00009	0.00009	0.00009	0.00009	0.001579579
PE(P-18:0/20:3)-H	0.006540205	0.003075952	0.005283562	0.009459028	0.003569554	0.009743134	0.001657965	0.008276329	0.011068832	0.007365656	0.002059121	0.001669529	0.003360485	0.008830962
PE(P-18:0/20:4)-H	0.096761296	0.056250949	0.097459822	0.168039366	0.056772042	0.168329912	0.035212661	0.123823658	0.147004682	0.115267617	0.033986738	0.033237418	0.044608901	0.153602634
PE(P-18:0/20:5)-H	0.001700977	0.001058306	0.001391269	0.002663167	0.000757305	0.002886755	0.000653515	0.003206171	0.003130485	0.002496252	0.000792178	0.000673288	0.001035222	0.003037963
PE(P-18:0/22:4)-H	0.003788726	0.002453567	0.004024273	0.006776309	0.001593447	0.006079475	0.00150354	0.004974143	0.004913649	0.004357785	0.001063759	0.000899692	0.001581165	0.005911847
PE(P-18:0/22:5)-H	0.008884231	0.004378069	0.006066768	0.009133962	0.003490046	0.009834628	0.002373562	0.00709048	0.014611575	0.013124686	0.002182419	0.00180923	0.00352524	0.018835829

PE(P-18:0/22:6)-H	0.08514955	0.045597161	0.086469324	0.084875151	0.049432817	0.082642575	0.030918321	0.049875201	0.129875163	0.131224772	0.011675906	0.011975739	0.039006424	0.17289682
PE(P-18:1/16:0)-H	0.025354995	0.012386872	0.022412879	0.028550274	0.013103822	0.026568808	0.01015548	0.020378074	0.033829574	0.029136219	0.005482479	0.005492254	0.010144859	0.039995016
PE(P-18:1/16:1)-H	0.002460976	0.001514048	0.002609831	0.003547772	0.001647916	0.003965533	0.000794098	0.002881701	0.004800016	0.004661749	0.000982495	0.000859013	0.001002908	0.004962856
PE(P-18:1/18:0)-H	0.00267299	0.001713282	0.002371726	0.003848096	0.001780618	0.00359687	0.001272764	0.002727634	0.004470605	0.004287764	0.000833572	0.000978349	0.001887072	0.005991101
PE(P-18:1/18:1)-H	0.026916285	0.015777379	0.023710774	0.044761793	0.015880499	0.042520651	0.011046632	0.04738508	0.059413282	0.041330347	0.011674548	0.012424184	0.018951082	0.065743241
PE(P-18:1/18:2)-H	0.017819178	0.012747256	0.017526352	0.029242532	0.012386329	0.028682278	0.008006188	0.02528287	0.044255575	0.04129589	0.006375607	0.007087074	0.012548261	0.055558564
PE(P-18:1/18:3)-H	0.00009	0.000665781	0.000756225	0.000682146	0.00009	0.000935027	0.00009	0.002489762	0.002053647	0.001420673	0.000625186	0.000486716	0.00069694	0.002131584
PE(P-18:1/20:1)-H	0.011982006	0.010198728	0.013030602	0.014670544	0.01124242	0.01525409	0.006247907	0.010303795	0.027880096	0.027771138	0.002511779	0.002720454	0.007848257	0.038516423
PE(P-18:1/20:2)-H	0.002408095	0.001772667	0.002136013	0.002119833	0.001865979	0.00288734	0.000929002	0.002167147	0.004548121	0.004164148	0.000450429	0.000643106	0.001324111	0.006460299
PE(P-18:1/20:3)-H	0.004251525	0.004003334	0.006022638	0.009117313	0.003825141	0.009355602	0.002378774	0.007763222	0.011436213	0.010423076	0.001949984	0.001508141	0.002865097	0.01329224
PE(P-18:1/20:4)-H	0.06410373	0.062930897	0.064449063	0.106551123	0.060947015	0.106517843	0.033710972	0.06271147	0.123819971	0.117173863	0.018127425	0.015811119	0.037021221	0.150184744
PE(P-18:1/20:5)-H	0.000863916	0.001057715	0.000886071	0.00229923	0.001033243	0.002162491	0.000324332	0.001600455	0.002580154	0.002166711	0.000483341	0.00009	0.001069975	0.002507001

PE(P-18:1/22:4)-H	0.001307924	0.001462177	0.001575322	0.002993832	0.000995428	0.002854027	0.000636583	0.001674754	0.003223748	0.002451203	0.000537522	0.000615598	0.000762639	0.00359885
PE(P-18:1/22:5)-H	0.005368791	0.004946916	0.005842725	0.007397309	0.005519013	0.007867708	0.002666903	0.005400848	0.014087621	0.014281028	0.001365821	0.001573023	0.003981849	0.016605777
PE(P-18:1/22:6)-H	0.079371829	0.069504448	0.082877114	0.10620234	0.070311048	0.107195096	0.040537806	0.058256384	0.180312333	0.189473118	0.019132697	0.015105531	0.055899102	0.234340428
PE(P-18:2/18:2)-H	0.005127671	0.004164358	0.005511075	0.008698607	0.003818591	0.007817729	0.002320584	0.006371789	0.01535226	0.013472166	0.001617869	0.001765977	0.004237763	0.018679929
PE(P-18:2/20:4)-H	0.016283872	0.014592165	0.01552043	0.02112313	0.015813022	0.021797948	0.008664593	0.013210612	0.029786336	0.030255315	0.002985669	0.003081866	0.008858282	0.037358552
PE(P-18:2/22:6)-H	0.022342972	0.016402526	0.023710344	0.027333511	0.018612472	0.02703851	0.009585898	0.013626569	0.051114357	0.051978139	0.003309296	0.003246755	0.015595858	0.068662895

Supplemental Table S2: TAG concentration (in µM) in EVs isolated from urine collected during the active phase (AP) compared to the inactive phase (IP)

Lipid	AP1	AP2	AP3	AP4	AP5	AP6	AP7	IP1	IP2	IP3	IP4	IP5	IP6	IP7
TAG(42:1/FA14:0)+NH4+A7:O81	0.042206505	0.037504831	0.046784272	0.03715215	0.02904709	0.00246	0.074383825	0.00246	0.25415162	0.00246	0.014715759	0.018567942	0.00246	0.024374278
TAG(46:4/FA18:2)+NH4	0.034969266	0.077996489	0.081880015	0.148517032	0.00246	0.145311483	0.090697933	0.205178388	0.056214662	0.019547441	0.044155136	0.019546442	0.00246	0.023091539
TAG(48:0/FA16:0)+NH4	0.00246	0.149372363	0.00246	0.00246	0.00246	0.52431253	0.00246	0.524014947	0.381727744	0.177954704	0.00246	0.098736129	0.62720876	0.137310329
TAG(48:0/FA18:0)+NH4	0.00246	0.00246	0.00246	0.00246	0.013700447	0.00246	0.00246	0.432269836	0.172480936	0.04104703	0.00246	0.00246	0.551882074	0.084687264
TAG(48:1/FA14:0)+NH4	0.00246	0.051683783	0.00246	0.00246	0.00246	0.00246	0.00246	0.00246	0.521283681	0.055717313	0.00246	0.039080575	0.172931209	0.00246
TAG(48:1/FA16:0)+NH4	0.00246	0.00246	0.00246	0.00246	0.00246	0.00246	0.00246	0.564069045	0.753598199	0.038610015	0.00246	0.00246	0.184276618	0.146288335
TAG(48:1/FA18:1)+NH4	0.074175736	0.049953732	0.00246	0.00246	0.00246	0.00246	0.00246	0.00246	0.929433538	0.044956438	0.00246	0.00246	0.174546819	0.09495163
TAG(48:2/FA18:1)+NH4	0.053671571	0.00246	0.00246	0.00246	0.00246	0.00246	0.00246	0.556771522	0.74340273	0.00246	0.00246	0.00246	0.066827894	0.00246
TAG(48:2/FA18:2)+NH4	0.033768087	0.026313828	0.00246	0.00246	0.00246	0.00246	0.00246	0.00246	0.504930257	0.00246	0.031277654	0.034208572	0.059408144	0.038489313
TAG(48:3/FA18:3)+NH4	0.00246	0.022330628	0.049375504	0.00246	0.00246	0.098343751	0.072564729	0.00246	0.218994431	0.00246	0.00246	0.012705375	0.00246	0.00246
TAG(48:4/FA14:0)+NH4	0.36273133	0.36599266	0.817336442	1.310074393	0.121773818	5.308943733	3.687948183	5.939173356	2.359684369	1.255980501	1.664538401	3.050254915	0.271895183	1.837359961
TAG(48:4/FA16:0)+NH4	0.02412254	0.015756551	0.03638157	0.037151431	0.00246	0.00246	0.00246	0.087922856	0.050233568	0.00246	0.00246	0.00246	0.00246	0.021808603
TAG(49:2/FA18:1)+NH4	0.00246	0.00246	0.11436497	0.245662834	0.00246	0.198396024	0.136080474	0.410277315	0.242737541	0.00246	0.074520761	0.00246	0.00246	0.00246
TAG(49:2/FA18:2)+NH4	0.00246	0.00246	0.080569332	0.00246	0.00246	0.096656633	0.00246	0.00246	0.070342804	0.00246	0.03495898	0.00246	0.052804929	0.016680774
TAG(49:3/FA18:2)+NH4	0.00246	0.00246	0.158557094	0.225679059	0.00246	0.195480143	0.152404358	0.512786087	0.202146001	0.00246	0.106725598	0.008790575	0.00246	0.00246
TAG(50:0/FA16:0)+NH4	0.065117812	0.105690671	0.00246	0.00246	0.040007146	0.176759567	0.00246	0.00246	0.409424685	0.109480244	0.00246	0.039102224	1.181990277	0.050681083

TAG(50:0/FA18:0)+NH4	0.088059805	0.042124017	0.00246	0.00246	0.029046449	0.147438434	0.090708448	0.45418293	0.545130075	0.058649184	0.0246	0.052772497	1.298380462	0.166842771
TAG(50:1/FA16:0)+NH4	0.196036398	0.146399039	0.132572411	0.077123347	0.056460856	0.163654118	0.00246	0.479879438	1.59013738	0.062553698	0.0246	0.034701969	0.487316356	0.228466023
TAG(50:1/FA16:1)+NH4	0.024120211	0.00246	0.00246	0.00246	0.00246	0.00246	0.00246	0.351679492	0.195436962	0.0246	0.0246	0.0246	0.104128485	0.0246
TAG(50:1/FA18:0)+NH4	0.03617768	0.00246	0.00246	0.00246	0.00246	0.00246	0.050783008	0.358958397	0.19066084	0.010750738	0.023915751	0.0246	0.103441285	0.038491654
TAG(50:1/FA18:1)+NH4	0.123628249	0.06965818	0.103967688	0.00246	0.00246	0.079901992	0.00246	0.571427286	0.526554079	0.0246	0.0246	0.0246	0.381979957	0.215664845
TAG(50:2/FA14:0)+NH4	0.0385904	0.040035241	0.00246	0.00246	0.00246	0.00246	0.00246	0.0246	0.854140249	0.034212756	0.0246	0.063492153	0.079664887	0.098179182
TAG(50:2/FA16:0)+NH4	0.724768914	0.207682614	0.338002055	0.131381279	0.111320601	0.193022823	0.156021152	0.219782185	2.817687431	0.106553045	0.0246	0.05669062	0.238902205	0.331895838
TAG(50:2/FA16:1)+NH4	0.00246	0.058840493	0.00246	0.00246	0.00246	0.00246	0.00246	0.336976496	1.821146943	0.0246	0.0246	0.0246	0.089402985	0.0246
TAG(50:2/FA18:1)+NH4	0.08443208	0.00246	0.094878436	0.077117611	0.00246	0.108202488	0.068931151	0.908603409	3.483388075	0.037137173	0.0246	0.0246	0.187082184	0.042980501
TAG(50:2/FA18:2)+NH4	0.146589525	0.075100325	0.088362115	0.00246	0.041665769	0.109874431	0.090721545	0.586077098	0.696693675	0.076253375	0.038638481	0.0246	0.097506747	0.168803085
TAG(50:3/FA16:0)+NH4	0.057889155	0.019143709	0.00246	0.00246	0.00246	0.00246	0.00246	0.0246	1.126275647	0.038117992	0.0246	0.014662077	0.055810877	0.07570143
TAG(50:3/FA18:2)+NH4	0.021707859	0.026798906	0.093583487	0.108540542	0.00246	0.124308837	0.076187232	0.1172131	1.169684766	0.02345377	0.0246	0.0246	0.074332871	0.0246
TAG(50:3/FA18:3)+NH4	0.019274539	0.018305993	0.00246	0.00246	0.00246	0.00246	0.00246	0.0246	0.193620489	0.005863443	0.0246	0.0246	0.029522304	0.03400031
TAG(51:0/FA16:0)+NH4	0.00246	0.00246	0.00246	0.00246	0.010413071	0.00246	0.00246	0.816981119	0.244675754	0.017590924	0.0246	0.0246	0.651951215	0.079561261
TAG(51:1/FA16:0)+NH4	0.00246	0.020422782	0.00246	0.00246	0.00246	0.00246	0.00246	0.490802661	0.234825007	0.0246	0.0246	0.01270562	0.434774802	0.021805864
TAG(51:1/FA18:1)+NH4	0.00246	0.00246	0.00246	0.00246	0.00246	0.00246	0.00246	0.446844427	0.226023343	0.0246	0.027598386	0.0246	0.48103299	0.032073786
TAG(51:2/FA17:0)+NH4	0.00246	0.009373126	0.00246	0.00246	0.00246	0.00246	0.00246	0.0246	0.083362912	0.009774591	0.0246	0.0246	0.06271998	0.012827205

TAG(51:2/FA18:1)+NH4	0.025325865	0.00246	0.00246	0.00246	0.009864023	0.00246	0.00246	0.00246	0.403866032	0.00246	0.00246	0.00246	0.08620135	0.037853583
TAG(52:0/FA16:0)+NH4	0.034969418	0.060610944	0.00246	0.057148875	0.00246	0.122709458	0.050793523	0.194133799	0.575990573	0.033233081	0.00246	0.041048005	1.197201814	0.05260963
TAG(52:0/FA18:0)+NH4	0.077179756	0.10103822	0.00246	0.054299069	0.018631846	0.202624517	0.068925989	0.512911149	0.224100787	0.018080662	0.034962346	0.058625032	3.503232013	0.097529795
TAG(52:1/FA16:0)+NH4	0.075378141	0.064754238	0.129962681	0.00246	0.013066541	0.039932897	0.076192217	0.68128296	0.404584706	0.065489966	0.034959728	0.00246	0.603240438	0.130889716
TAG(52:1/FA18:0)+NH4	0.072359344	0.097874103	0.064977179	0.057151575	0.036175765	0.00246	0.00246	0.388297771	0.365235731	0.10166088	0.00246	0.033227736	0.932754859	0.051317952
TAG(52:1/FA18:1)+NH4	0.050656392	0.039564996	0.00246	0.00246	0.013153377	0.00246	0.072555319	0.32963505	0.690645175	0.05864342	0.00246	0.029319838	1.670380226	0.109076218
TAG(52:1/FA20:0)+NH4	0.00246	0.00246	0.00246	0.00246	0.00246	0.00246	0.00246	0.20516608	0.135832722	0.00246	0.00246	0.00246	0.057581565	0.00246
TAG(52:2/FA16:0)+NH4	0.269153623	0.283458882	0.22095481	0.142831344	0.121781097	0.249750571	0.130623005	0.556778829	2.407485318	0.127100933	0.00246	0.06451208	0.473967107	0.20791074
TAG(52:2/FA16:1)+NH4	0.00246	0.00246	0.00246	0.00246	0.00246	0.00246	0.00246	0.651935719	0.468171601	0.00246	0.00246	0.00246	0.090140296	0.00246
TAG(52:2/FA18:0)+NH4	0.261898113	0.060257164	0.093574368	0.019987954	0.024115384	0.050285679	0.029018437	0.351589063	0.515213681	0.045939891	0.00246	0.00246	0.190551268	0.177116106
TAG(52:2/FA18:1)+NH4	0.533165236	0.227461754	0.285969247	0.217122895	0.041105632	0.281506917	0.174181656	0.652090614	4.44658473	0.135873338	0.034968054	0.04497035	0.441328789	0.368598353
TAG(52:2/FA18:2)+NH4	0.1918388	0.1210137	0.046793126	0.048548651	0.009863522	0.156722521	0.094333355	0.131858767	0.654963147	0.037137173	0.00246	0.00246	0.195841682	0.237525893
TAG(52:3/FA16:0)+NH4	0.260076878	0.385517287	0.090966161	0.108528786	0.184980383	0.529594133	0.279471084	0.315017633	2.272719348	0.59901649	0.040481389	0.034214032	0.19841773	0.312021029
TAG(52:3/FA18:1)+NH4	0.391281994	0.20437048	0.7443718	0.305726287	0.123951346	0.390165158	0.206833672	0.355250475	5.490418934	0.358297726	0.033116118	0.055724582	0.279135093	0.446509759

TAG(52:3/FA18:2)+NH4	0.376577205	0.207423152	0.421317366	0.128541419	0.115210776	0.384077772	0.399294825	0.406568651	4.111049065	0.21416706	0.040313763	0.023714971	0.190414607	1.124538355
TAG(52:3/FA18:3)+NH4	0.026526431	0.020860477	0.00246	0.017137636	0.007125026	0.00246	0.00246	0.00246	0.111894353	0.011726985	0.00246	0.00246	0.061033032	0.029510654
TAG(52:4/FA16:0)+NH4	0.797273962	0.721208107	0.408312998	0.411500164	0.191047271	0.68711998	0.087085017	0.380977555	1.820061188	0.60290488	0.00246	0.010753088	0.164314155	0.814915879
TAG(52:4/FA18:1)+NH4	0.088055851	0.042360947	0.059780317	0.065690793	0.00246	0.045660497	0.050807357	0.175783153	2.518441111	0.092879183	0.00246	0.00246	0.064026736	0.047472342
TAG(52:4/FA18:2)+NH4	0.909639721	0.513680914	0.598416095	0.322840721	0.243332059	0.515801643	0.453792763	0.575101015	3.350356212	1.469976074	0.079119823	0.034217162	0.156441845	0.657891466
TAG(52:4/FA18:3)+NH4	0.088049842	0.0876997	0.090970851	0.085677002	0.014250386	0.124832255	0.061682076	0.00246	0.691625277	0.124181138	0.02372639	0.006841166	0.083783141	0.088536984
TAG(52:5/FA16:0)+NH4	0.142359555	0.116969449	0.097410122	0.088541993	0.059764756	0.216541438	0.112488327	0.146469449	0.259125935	0.110489437	0.00246	0.00246	0.064427826	0.102664085
TAG(52:5/FA18:2)+NH4	0.187056952	0.080817378	0.054576326	0.085680488	0.038372736	0.130803526	0.00246	0.036621651	1.426595789	0.123215625	0.018059155	0.022478817	0.048511669	0.09367362
TAG(52:5/FA18:3)+NH4	0.107349333	0.208468848	0.163775231	0.185682988	0.038921784	0.177776242	0.039912344	0.252710985	0.624797032	0.131525124	0.025759735	0.029329024	0.07259965	0.127045343
TAG(52:5/FA20:5)+NH4	0.033771826	0.038748598	0.057181428	0.044254013	0.006028519	0.00246	0.00246	0.805895262	0.378404102	0.00246	0.00246	0.00246	0.114310214	0.03079222
TAG(52:5/FA22:5)+NH4	0.048234168	0.057779166	0.00246	0.00246	0.00246	0.057088003	0.00246	0.754674833	0.291900074	0.012705865	0.00246	0.021499769	0.085861519	0.00246
TAG(52:6/FA18:3)+NH4	0.02532513	0.038838411	0.054594953	0.0354676	0.00246	0.00246	0.00246	0.00246	0.309941665	0.029322779	0.00246	0.00246	0.00246	0.00246

TAG(54:1/FA18:0)+NH4	0.039795686	0.049683622	0.00246	0.048553584	0.027951029	0.04992361	0.065303664	0.183155504	0.114978005	0.052298511	0.00246	0.00246	0.995948556	0.089817377
TAG(54:1/FA18:1)+NH4	0.013265515	0.031914352	0.01819713	0.020009294	0.007125082	0.039933404	0.00246	0.190486526	0.173289137	0.00246	0.011039018	0.00246	0.792182434	0.024376693
TAG(54:1/FA20:0)+NH4	0.00246	0.024106313	0.070173109	0.045698044	0.00246	0.108548295	0.097981504	0.593425992	0.337289853	0.00246	0.00246	0.00246	0.172212408	0.017961688
TAG(54:2/FA18:0)+NH4	0.078394487	0.076660684	0.176771011	0.082838293	0.021924404	0.088820502	0.090708818	0.00246	0.242684427	0.08749154	0.00246	0.00246	0.223037526	0.109075503
TAG(54:2/FA18:1)+NH4	0.161626121	0.136105269	0.137750371	0.134258891	0.02027818	0.00246	0.00246	0.417538909	0.761416123	0.09091372	0.00246	0.036656464	0.436182464	0.224658439
TAG(54:2/FA18:2)+NH4	0.025322125	0.029788411	0.020804332	0.028558515	0.004933488	0.00246	0.021768994	0.00246	0.094067223	0.00246	0.00246	0.00246	0.08990463	0.053887608
TAG(54:2/FA20:0)+NH4	0.022912165	0.007876628	0.023389892	0.00246	0.00246	0.053274256	0.00246	0.00246	0.258922354	0.00246	0.00246	0.00246	0.070938678	0.019243775
TAG(54:3/FA18:0)+NH4	0.13029197	0.151667718	0.103969008	0.060257446	0.052080149	0.08418427	0.105229105	0.161114418	0.356276735	0.239715684	0.018401482	0.005864652	0.075485495	0.328798542
TAG(54:3/FA18:1)+NH4	0.630055937	0.514729826	0.280815872	0.328596687	0.242761249	0.183846306	0.00246	0.41024063	2.485286781	0.132478514	0.057042348	0.334856186	0.183045903	0.762877889
TAG(54:3/FA18:2)+NH4	0.255857637	0.088211095	0.192368323	0.062837996	0.03124002	0.07561842	0.076198488	0.190434423	0.514099801	0.153530302	0.027597264	0.026389646	0.041248002	0.093681839
TAG(54:4/FA18:0)+NH4	0.278258817	0.195701908	0.197582623	0.039982155	0.067985564	0.219746949	0.00246	0.00246	0.3878655	0.053759851	0.00246	0.00246	0.064359507	0.112936638
TAG(54:4/FA18:1)+NH4	1.144538394	0.409722388	0.447373939	0.00246	0.165776194	0.913147578	0.130634259	0.871939675	2.985970029	0.433279327	0.00246	0.391740637	0.08981056	0.98321451

TAG(54:4/FA18:2)+NH4	0.474714267	0.386444317	0.233991158	0.257126967	0.18829019	0.549522059	0.00246	0.509157862	3.067184676	0.065495878	0.00246	0.109047238	0.139680655	1.07540856
TAG(54:5/FA18:1)+NH4	0.266664577	0.562955826	0.189748016	0.32000507	0.116817181	0.542070675	0.119750499	0.344262892	2.570180614	0.389259335	0.046002769	0.053768622	0.182377225	1.40107592
TAG(54:5/FA18:2)+NH4	0.78467332	0.697105284	0.692255018	0.815059213	0.2288378	0.638406261	0.195951019	0.424851917	1.9538097	0.776828735	0.060723112	0.075278776	0.117026498	0.326090861
TAG(54:5/FA18:3)+NH4	0.154402554	0.068315884	0.109169304	0.00246	0.019180364	0.00246	0.083450701	0.00246	0.449701729	0.07918291	0.00246	0.031245464	0.046483011	0.00246
TAG(54:6/FA18:1)+NH4	0.214765924	0.120793579	0.158582205	0.074265994	0.081169982	0.159834568	0.126998837	0.139181988	0.769550111	0.185854615	0.00246	0.039107187	0.024343303	0.343006723
TAG(54:6/FA18:2)+NH4	1.070622967	1.736703855	1.292085722	0.454427328	0.426334348	1.401665555	0.748138898	0.249050669	2.227506262	2.026277649	0.060723486	0.100719341	0.218678668	3.160208465
TAG(54:6/FA18:3)+NH4	0.070558802	0.217002792	0.168976054	0.00246	0.09434189	0.172555873	0.195975001	0.00246	0.472013273	0.087010275	0.00246	0.025414073	0.04704108	0.169452803
TAG(54:7/FA18:2)+NH4	0.408206012	0.366392972	0.332827135	0.142822057	0.065248824	0.29339437	0.170545866	0.197772533	0.822925595	0.276005953	0.038634973	0.012703138	0.063388292	0.179738925
TAG(54:7/FA18:3)+NH4	0.182203922	0.089945207	0.214495507	0.142822943	0.048245091	0.333196609	0.166934425	0.227001361	0.564750172	0.010750639	0.00246	0.00246	0.062728577	0.187422189
TAG(54:8/FA18:2)+NH4	0.009643768	0.029331062	0.119568697	0.085698773	0.00822	0.119367135	0.00246	0.102546192	0.239984317	0.012703032	0.016559366	0.00246	0.00246	0.00246
TAG(54:8/FA18:3)+NH4	0.162890452	0.054935084	0.00246	0.077125021	0.00246	0.044938026	0.00246	0.109851178	0.08826515	0.087015443	0.00246	0.010749362	0.02640003	0.096252146

Supplemental Table S3: PC and LPC concentration (in µM) in EVs isolated from urine collected during the active phase (AP) compared to the inactive phase (IP)

Lipid	AP1	AP2	AP3	AP4	AP5	AP6	AP7	IP1	IP2	IP3	IP4	IP5	IP6	IP7
PC(14:0/20:4)+AcO	0.012448064	0.015764512	0.016087322	0.014139328	0.013773047	0.015170672	0.0018	0.0018	0.029439256	0.020161499	0.0018	0.0018	0.011288767	0.024607099
PC(14:0/22:6)+AcO	0.177029208	0.103415241	0.12518894	0.179062937	0.155883638	0.133369221	0.085522632	0.075899197	0.297867617	0.234794933	0.016510216	0.014638345	0.080840348	0.293814054
PC(16:0/18:0)+AcO	0.142176477	0.066156695	0.098973538	0.109495215	0.092496496	0.104901346	0.042419055	0.0018	0.319948112	0.266392588	0.0018	0.0018	0.0018	0.327329744
PC(16:0/20:4)+AcO	0.010382937	0.01033265	0.010623025	0.014250485	0.010188555	0.027979053	0.003641654	0.040648773	0.060560194	0.017790497	0.008169544	0.007234204	0.014199956	0.015935212
PC(16:0/22:6)+AcO	0.050075379	0.021785219	0.035860538	0.039983952	0.035501497	0.051922609	0.011093175	0.033596315	0.084674122	0.091422641	0.005995194	0.008052587	0.028654325	0.074051362
PC(18:0/18:0)+AcO	0.172918245	0.077851129	0.110663868	0.302938182	0.107588993	0.289305712	0.067318883	0.394840242	0.387093972	0.270304922	0.078054732	0.073279772	0.076286534	0.237105385
PC(18:0/18:1)+AcO	0.046535508	0.036957988	0.031205894	0.105827674	0.040112138	0.083387803	0.025976278	0.499346315	0.568602366	0.073578513	0.117909205	0.08396897	3.798853274	0.181682201
PC(18:0/20:0)+AcO	0.52082139	0.296119446	0.331046869	0.425581041	0.434179992	0.375087723	0.205716562	0.18837481	0.876191421	1.26236084	0.060918811	0.060750122	0.3107668	1.082114362
PC(18:0/20:4)+AcO	0.02061593	0.009035769	0.011868316	0.021733616	0.010497077	0.025488501	0.005729073	0.033935317	0.031343478	0.022290554	0.00829251	0.0018	0.026555911	0.023302976
PC(18:1/18:1)+AcO	0.038488635	0.047058869	0.025475903	0.072567991	0.060981734	0.096333988	0.015382224	0.690704447	0.503535342	0.202056986	0.151673074	0.128516257	0.662306135	0.251021176
PC(18:1/18:2)+AcO	0.011713896	0.014468624	0.012461732	0.031775618	0.01606262	0.026149643	0.005663314	0.166640515	0.226342504	0.046080144	0.038724926	0.0289726	0.08369191	0.072316012
PC(18:1/18:3)+AcO	0.0018	0.0018	0.0018	0.0018	0.0018	0.0018	0.0018	0.041751136	0.030950102	0.009829775	0.007797073	0.006033538	0.01046823	0.012284967
PC(18:2/18:2)+AcO	0.013149258	0.012736874	0.012195876	0.009575549	0.005567414	0.00948538	0.004103188	0.071252163	0.095799293	0.029150793	0.019801682	0.016081171	0.009282489	0.024957271
LPC(16:0)+AcO	0.006783422	0.132009984	0.068368795	0.036444177	0.024994913	0.028719193	0.023824558	0.343556208	0.492921545	0.011806724	0.026871138	0.016643027	0.904628455	0.023875301
LPC(16:1)+AcO	0.001301828	0.035851045	0.015780058	0.006674708	0.00664613	0.004483291	0.004288035	0.003344658	0.031029158	0.003022916	0.004781335	0.005836098	0.039505306	0.001212223
LPC(18:0)+AcO	0.014430663	0.039399199	0.041554765	0.034356846	0.02328149	0.033479386	0.013921873	0.2345134	0.280913651	0.018471273	0.017337616	0.023521544	0.02	0.032237848

LPC(18:1)+AcO	0.004670114	0.10633125	0.048581125	0.018083022	0.023003996	0.016257456	0.012660896	0.017770502	0.159500416	0.011339246	0.010578885	0.016022236	0.592856303	0.012231003
LPC(18:2)+AcO	0.008690499	0.028568036	0.017745922	0.013347203	0.015541006	0.015542213	0.012284203	0.020655728	0.037056548	0.012199786	0.015474585	0.011396891	0.138235199	0.012486885
LPC(20:0)+AcO	0.001055316	0.005648915	0.003830102	0.003511029	0.003491524	0.002396722	0.001839379	0.020954284	0.029087469	0.002910146	0.0005	0.0005	0.259028758	0.006228136
LPC(20:4)+AcO	0.012340031	0.025497101	0.026945544	0.018696681	0.021120234	0.018434145	0.015362261	0.02145699	0.023219172	0.017070949	0.019365109	0.016445473	0.021402203	0.015506212
LPC(20:5)+AcO	0.004552527	0.005356853	0.005929727	0.006303536	0.006811798	0.007725101	0.004002354	0.008274521	0.006395554	0.005656651	0.007471695	0.005678424	0.006988612	0.00594732

Supplemental Table S4: PS concentration (in μM) in EVs isolated from urine collected during the active phase (AP) compared to the inactive phase (IP)

Lipid	AP1	AP2	AP3	AP4	AP5	AP6	AP7	IP1	IP2	IP3	IP4	IP5	IP6	IP7
PS(16:0/16:1)-H	0.001526521	0.000735661	0.001080104	0.001138025	0.000534333	0.001176447	0.000427315	0.000627552	0.001426781	0.000985448	0.0006	0.0012855	0.000426983	0.00171423
PS(16:0/18:0)-H	0.00207976	0.001244897	0.001872763	0.002563483	0.00090693	0.002037612	0.000718491	0.001477806	0.00315301	0.002972461	0.000436493	0.000364495	0.00114446	0.004118637
PS(16:0/20:4)-H	0.000729704	0.00058456	0.000475182	0.001718816	0.000529087	0.002019341	0.00006	0.001399328	0.00173262	0.001238253	0.000499615	0.000283421	0.000366847	0.000945886
PS(18:0/18:0)-H	0.031779716	0.019431989	0.029121354	0.046331262	0.019137024	0.049316994	0.01283763	0.039278146	0.045836338	0.035646013	0.011756741	0.010207614	0.013538062	0.045209456
PS(18:0/18:1)-H	0.007490043	0.006009723	0.006161962	0.015080343	0.005118235	0.016123911	0.00334451	0.015264419	0.017532695	0.014110638	0.003626662	0.003646632	0.004850043	0.017396544
PS(18:0/18:2)-H	0.001124407	0.001126473	0.001115939	0.001379434	0.001151764	0.001543437	0.00049061	0.001209312	0.001809368	0.002107475	0.000327756	0.0006	0.000750288	0.002036634
PS(18:0/20:4)-H	0.001188297	0.000818633	0.001060254	0.002258034	0.000589917	0.00180344	0.000555734	0.001165454	0.001823478	0.001608187	0.00057662	0.00042227	0.000590675	0.001440331
PS(18:0/22:6)-H	0.001847926	0.000881311	0.001540695	0.00159212	0.001100374	0.001456228	0.000517102	0.000598847	0.00205375	0.002516815	0.0006	0.0006	0.000692212	0.002016015
PS(18:1/18:2)-H	0.000482908	0.000582653	0.00006	0.001016325	0.00042911	0.000836193	0.00006	0.000650295	0.001398228	0.000854489	0.0006	0.000264991	0.000455828	0.001128229
PS(18:1/20:4)-H	0.003784218	0.003148922	0.003770702	0.007380265	0.002810775	0.007688376	0.001519358	0.004941895	0.006047354	0.004418986	0.001426213	0.001620916	0.002051468	0.006428355
PS(18:1/22:6)-H	0.006247565	0.003420778	0.005533981	0.005855477	0.003709694	0.006598484	0.002717355	0.003832808	0.008268327	0.010063375	0.000790704	0.001184962	0.003130353	0.011780011

Supplemental Table S5: SM concentration (in μM) in EVs isolated from urine collected during the active phase (AP) compared to the inactive phase (IP)

Lipid	AP1	AP2	AP3	AP4	AP5	AP6	AP7	IP1	IP2	IP3	IP4	IP5	IP6	IP7
SM(14:0)+H	0.034921323	0.066148242	0.048005623	0.042625607	0.019875543	0.044428752	0.01528002	0.15610147	0.139242696	0.076875818	0.032334766	0.030750732	0.356278531	0.093198653
SM(16:0)+H	1.609162191	0.977194013	1.512423402	1.404511017	0.712993466	1.736348607	0.658085452	1.897455931	2.262472754	1.515254196	0.457688061	0.443439183	4.120423563	1.956438982
SM(18:0)+H	0.022322934	0.016823644	0.022560863	0.026085558	0.010532121	0.029693581	0.008189775	0.059460241	0.059675939	0.03042142	0.014714413	0.013793454	0.457696867	0.043250869
SM(18:1)+H	0.003841342	0.003633557	0.004069712	0.004628269	0.002181204	0.005903271	0.002077838	0.009889038	0.010152551	0.006825888	0.002844176	0.003147012	0.050016831	0.007636668
SM(20:0)+H	0.074866457	0.047220065	0.069075742	0.101855187	0.033497139	0.119898611	0.03133892	0.251475487	0.236811029	0.134727002	0.058513786	0.056101222	0.10810222	0.163988993
SM(20:1)+H	0.004737483	0.00699392	0.006362959	0.00595779	0.002442329	0.007737743	0.00237094	0.008450522	0.01113613	0.00709999	0.002657356	0.002808432	0.006169588	0.008448619
SM(22:0)+H	0.474393062	0.253077176	0.410496676	0.629355015	0.21075976	0.654503962	0.176087642	1.019274772	1.008092123	0.628883261	0.273322589	0.255262773	0.773976162	0.805827037
SM(22:1)+H	0.097913065	0.090231658	0.102059475	0.114736789	0.048663586	0.125690149	0.041090532	0.184361613	0.197095382	0.12450774	0.049954947	0.046943137	0.154811573	0.154789627
SM(24:0)+H	0.454929458	0.258305957	0.430931175	0.62571027	0.234045487	0.628009546	0.199228604	0.783950129	0.812233218	0.532165409	0.198411121	0.19369971	0.918824039	0.71250859
SM(24:1)+H	0.495809387	0.293962121	0.45596311	0.568136869	0.248058397	0.651691083	0.211761934	0.642962313	0.743021511	0.489326045	0.162069756	0.15826377	0.62997963	0.631358187
SM(26:0)+H	0.004076397	0.00319792	0.003678619	0.005523479	0.002119487	0.006053231	0.001815218	0.012144568	0.010156873	0.00583491	0.002701668	0.002702744	0.025296036	0.007689784
SM(26:1)+H	0.00147832	0.001296117	0.00162042	0.002089341	0.000902562	0.002476126	0.000696514	0.003875079	0.003668739	0.002123562	0.000933256	0.000878651	0.009405535	0.002825633

Supplemental Table S6: CER, DCER, HCER concentration (in μM) in EVs isolated from urine collected during the active phase (AP) compared to the inactive phase (IP)

Lipid	AP1	AP2	AP3	AP4	AP5	AP6	AP7	IP1	IP2	IP3	IP4	IP5	IP6	IP7
CER(16:0)+H	0.0048	0.0054	0.0038	0.0035	0.0056	0.0053	0.0022	0.0052	0.0058	0.0045	0.0026	0.0027	1E-05	0.0054
CER(18:0)+H	0.0035	0.0011	0.0009	0.0012	0.0014	0.002	1E-05	0.0017	0.0032	0.0035	1E-05	1E-05	0.0027	0.0038
CER(18:1)+H	0.0124	0.0068	0.0039	0.0041	0.0072	0.0044	1E-05	0.0024	0.0121	0.0113	1E-05	0.0009	0.0026	0.0137
CER(20:1)+H	0.0069	0.0052	0.0033	0.0023	0.0045	0.0034	0.0014	0.0029	0.0093	0.009	0.0007	0.0013	0.0025	0.0088
CER(22:1)+H	0.0108	0.007	0.0055	0.0071	0.0062	0.0087	0.0042	0.0031	0.0165	0.0149	0.0015	1E-05	0.0044	0.0268
CER(24:0)+H	0.0068	0.0039	1E-05	0.0033	0.0026	0.0038	0.002	0.0041	0.0056	0.0041	0.0015	0.0016	0.0062	0.0055
CER(24:1)+H	0.0189	0.0185	0.0114	0.0084	0.0156	0.0128	0.0097	0.012	0.0249	0.0246	0.0024	0.0032	0.0127	0.0242
CER(26:1)+H	0.013	0.0139	0.0075	0.01	0.0163	0.0115	0.0102	0.0056	0.0231	0.0206	0.0023	0.0031	0.008	0.0261
DCER(14:0)+H	0.0119	0.015	0.0116	0.009	0.0164	0.0089	0.0117	0.0098	0.0142	0.014	0.01	0.0147	0.0133	0.0152
DCER(16:0)+H	0.0089	0.0097	0.006	0.0054	0.0073	0.0064	0.0022	0.0066	0.0153	0.0126	0.0018	0.0023	0.0082	0.0182
DCER(18:0)+H	0.0022	0.0017	0.0012	0.002	0.001	0.0024	0.0012	0.002	0.0028	0.0029	1E-05	0.0008	0.0014	0.003
DCER(18:1)+H	0.0014	0.0014	0.0014	0.0014	0.0011	1E-05	0.0007	0.0018	0.0029	0.0018	1E-05	1E-05	0.0016	0.003
DCER(20:0)+H	1E-05	1E-05	1E-05	1E-05	0.0017	0.006	0.0019	0.0039	0.0025	1E-05	0.0023	1E-05	0.0011	0.0034
DCER(24:0)+H	0.0015	0.0018	0.0169	0.0172	0.001	0.0166	0.0119	0.0182	0.0039	0.0023	0.0142	1E-05	0.0044	0.0036
HCER(16:0)+H	0.0066	0.0045	0.0061	0.0054	0.0033	0.0065	0.0025	0.0037	0.0077	0.0069	0.0009	0.001	0.0029	0.0098
HCER(18:0)+H	0.0002	0.0002	0.0002	0.0003	0.0001	0.0002	8E-05	0.0003	0.0005	0.0003	4E-05	1E-04	0.0003	0.0004
HCER(18:1)+H	0.0054	0.0038	0.0046	0.0051	0.0029	0.0062	0.0021	0.0074	0.0088	0.0063	0.0018	0.0019	0.0058	0.0086
HCER(20:0)+H	0.0011	0.0005	0.0007	0.0011	0.0004	0.0012	0.0003	0.0011	0.0013	0.0008	0.0002	0.0002	0.0004	0.0012
HCER(20:1)+H	0.0174	0.01	0.0156	0.0205	0.0075	0.0234	0.006	0.0175	0.0219	0.0168	0.0044	0.0042	0.007	0.0223
HCER(22:0)+H	0.0026	0.0015	0.0022	0.0031	0.0012	0.0036	0.0008	0.0034	0.0037	0.0024	0.0007	0.0007	0.0016	0.0034
HCER(22:1)+H	0.0433	0.0265	0.038	0.0528	0.0195	0.0602	0.0154	0.055	0.0667	0.0444	0.0143	0.0145	0.0266	0.0615
HCER(24:0)+H	0.0025	0.0014	0.0019	0.003	0.001	0.003	0.0013	0.0036	0.004	0.0025	0.0009	0.001	0.0025	0.0036
HCER(24:1)+H	0.0353	0.0204	0.0307	0.0408	0.017	0.0451	0.0132	0.0529	0.0617	0.04	0.0139	0.014	0.0336	0.0564
HCER(26:1)+H	0.0016	0.0012	0.0014	0.0023	0.001	0.0022	0.0008	0.0028	0.0026	0.0015	0.0006	0.0007	0.0017	0.0022

***Supplemental Table S7**:* PG concentration (in μM) in EVs isolated from urine collected during the active phase (AP) compared to the inactive phase (IP)

Lipid	AP1	AP2	AP3	AP4	AP5	AP6	AP7	IP1	IP2	IP3	IP4	IP5	IP6	IP7
PG(14:0/14:0)-H	0.004664412	0.000453611	0.004560965	0.004248924	0.000624512	0.004067183	0.00128564	0.001924029	0.005005457	0.00440391	0.000278939	0.000256036	0.00104726	0.006264062
PG(14:0/18:1)-H	0.017362755	0.014167778	0.01740357	0.087623504	0.015630465	0.08906619	0.007729565	0.027881627	0.086819838	0.078570198	0.005269225	0.004432617	0.020505374	0.110768153
PG(14:0/18:2)-H	0.017097875	0.005149951	0.017675079	0.049067006	0.005953521	0.046557316	0.005917104	0.012612021	0.040420997	0.036057865	0.002240428	0.002217459	0.009727815	0.051102226
PG(14:0/18:3)-H	0.001737907	0.000614374	0.001864176	0.00342728	0.000504563	0.003572897	0.000496462	0.001289035	0.003488756	0.003065662	0.000256155	0.000179639	0.000886083	0.004200815
PG(14:0/20:1)-H	0.001150148	0.000465419	0.001044567	0.00245576	0.000658713	0.002455892	0.000383035	0.000418133	0.001930346	0.002100791	0.000046	0.000113662	0.000450178	0.002647615
PG(14:0/20:2)-H	0.000229171	0.000046	0.000046	0.001467579	0.000046	0.001462736	0.000046	0.000176817	0.000486306	0.00036276	0.000046	0.000046	0.000046	0.000557543
PG(16:0/14:0)-H	0.080482909	0.038984709	0.083006405	0.243887158	0.045638475	0.241184075	0.028245309	0.085641975	0.258948727	0.223307197	0.014292124	0.01266403	0.056589534	0.313262358
PG(16:0/16:0)-H	0.028724505	0.034343731	0.030372064	0.104403923	0.039045559	0.10381624	0.015168282	0.066218872	0.142754479	0.122231066	0.012877224	0.012470518	0.034104317	0.166076149
PG(16:0/16:1)-H	0.009076085	0.004614292	0.009695364	0.024270654	0.004970857	0.024245892	0.003545374	0.010367398	0.024366889	0.02152381	0.001846188	0.001881797	0.0053569	0.029288688
PG(16:0/18:0)-H	0.002548146	0.002144706	0.002486637	0.007590789	0.002357104	0.007599053	0.001194041	0.003646291	0.007591986	0.00661891	0.000780437	0.000808897	0.002060742	0.008588553
PG(16:0/18:1)-H	0.037048857	0.041055548	0.038750999	0.098740821	0.046602104	0.099429143	0.021232286	0.052149394	0.131892911	0.121124517	0.011638367	0.011781435	0.035568652	0.16169392
PG(16:0/18:2)-H	0.057522965	0.026465725	0.061832312	0.078956312	0.029163127	0.079824307	0.021232839	0.043127943	0.081548962	0.067375452	0.009155376	0.008673693	0.020853439	0.092914561
PG(16:0/18:3)-H	0.008834125	0.004371923	0.008757452	0.011238313	0.004932836	0.011202264	0.003181308	0.007034277	0.01222768	0.010613944	0.001377248	0.001471631	0.003140792	0.013761765
PG(16:0/20:1)-H	0.003857537	0.005368147	0.003782537	0.004162851	0.006066736	0.004194117	0.002286928	0.002142481	0.007079529	0.00649866	0.000460975	0.000424205	0.001870871	0.008964616
PG(16:0/20:2)-H	0.000629518	0.000502438	0.000668071	0.001626167	0.000505926	0.001409532	0.000277547	0.000728165	0.001349139	0.001085456	0.000146853	0.000173852	0.000313005	0.001387093
PG(16:0/20:3)-H	0.000569115	0.000046	0.000581158	0.000567565	0.000046	0.00055099	0.000046	0.000046	0.000349553	0.000046	0.000046	0.000046	0.000046	0.000354594

PG(18:0/14:0)-H	0.011212866	0.005127172	0.011220489	0.05420757	0.005549552	0.053258141	0.003913338	0.01235709	0.034337596	0.031222174	0.002301655	0.002004284	0.008535482	0.043325736
PG(18:0/16:1)-H	0.006410487	0.001786409	0.006977286	0.007479547	0.002042361	0.007233994	0.002112172	0.002625989	0.008840365	0.008661662	0.000553853	0.000486315	0.002170572	0.011251249
PG(18:0/18:0)-H	0.000929692	0.000741373	0.001023926	0.00162902	0.000758825	0.001431577	0.000503435	0.000868684	0.003558067	0.003241199	0.000046	0.000046	0.00089735	0.004652672
PG(18:0/18:1)-H	0.01724012	0.014731253	0.016902264	0.033791684	0.016418925	0.032165948	0.008739145	0.016665264	0.067916138	0.067920478	0.003768973	0.00381012	0.018711314	0.090817258
PG(18:0/18:2)-H	0.03358802	0.009565096	0.035760955	0.027690515	0.01075395	0.027622425	0.011517957	0.015052503	0.026941626	0.022710055	0.00327017	0.002993782	0.007364658	0.031119699
PG(18:0/18:3)-H	0.005504142	0.001531168	0.00554611	0.004183056	0.001767153	0.004373513	0.001757422	0.002783031	0.004021164	0.003137959	0.000530214	0.000552198	0.001134099	0.004173752
PG(18:0/20:1)-H	0.00161792	0.00184735	0.001537154	0.001069717	0.002184241	0.00109013	0.000839701	0.000679906	0.00228269	0.001910601	0.00017063	0.000143744	0.000643638	0.002912815
PG(18:0/20:2)-H	0.000293225	0.000141656	0.000249937	0.000288245	0.000147528	0.000296372	9.14967E-05	0.000210461	0.000318468	0.000295985	0.000046	0.000046	9.73531E-05	0.000385948
PG(18:1/16:1)-H	0.012365246	0.0077424	0.013889271	0.035374248	0.009579103	0.036029773	0.005316593	0.009691391	0.067456277	0.067936485	0.002092825	0.00173725	0.016920729	0.091332456
PG(18:1/18:1)-H	0.078210735	0.068116544	0.079222368	0.221419514	0.078307646	0.223201546	0.038399835	0.068147635	0.416107801	0.424375235	0.016346591	0.015358405	0.116788975	0.576767917
PG(18:1/18:2)-H	0.102148065	0.029323572	0.103915528	0.100130169	0.0341981	0.102380215	0.033761927	0.050439324	0.114227691	0.101229546	0.010659032	0.010184768	0.030807472	0.136427474
PG(18:1/18:3)-H	0.014732136	0.004580116	0.015023087	0.013807554	0.005426872	0.013864278	0.004954803	0.009497147	0.01672634	0.014104212	0.002001612	0.001802352	0.004607593	0.018749698
PG(18:1/20:1)-H	0.005312894	0.005141324	0.005285511	0.004882914	0.006212513	0.005159488	0.002737488	0.00294619	0.009632002	0.008966561	0.000646979	0.000590526	0.002783734	0.012863439
PG(18:1/20:2)-H	0.000432127	0.000288066	0.000531561	0.000508716	0.000355966	0.000509584	0.000108488	0.000317134	0.00085866	0.000987944	0.000046	0.000046	0.000191988	0.001071935
PG(18:2/16:1)-H	0.017700395	0.002976082	0.019100005	0.017850286	0.003429403	0.017189945	0.005228781	0.006414523	0.017678018	0.01640105	0.00119601	0.001241255	0.004471098	0.021300347
PG(18:2/18:2)-H	0.251600747	0.021599636	0.261440172	0.144886671	0.024927693	0.143842467	0.073005476	0.073573998	0.106761017	0.087025977	0.014744498	0.014502297	0.028426337	0.113097768
PG(18:2/18:3)-H	0.030298259	0.002905288	0.031596985	0.017310132	0.003477364	0.018583589	0.008477237	0.010814082	0.015689427	0.012542196	0.002419211	0.002311084	0.004156206	0.016653569

PG(18:2/20:1)-H	0.011035339	0.003326721	0.011875311	0.008286724	0.004231322	0.008343957	0.003817268	0.00298673	0.007979816	0.007334884	0.000651898	0.000632007	0.002130756	0.009829763
PG(18:2/20:2)-H	0.000827901	0.000192234	0.001008262	0.000534143	0.000230274	0.000681363	0.000317332	0.000372612	0.00065857	0.000652911	0.000100888	0.000046	0.000172545	0.00072917
PG(20:0/16:1)-H	0.001069086	0.000213661	0.000941815	0.000444616	0.00028363	0.000453727	0.000441742	0.000046	0.000708256	0.000747885	0.000046	0.000046	0.000169848	0.000761045
PG(20:0/18:1)-H	0.001803739	0.001350999	0.001527501	0.001433087	0.001591462	0.00144987	0.000836736	0.000852682	0.003623724	0.003018044	0.000267996	0.000115595	0.00099366	0.00463734
PG(20:0/18:2)-H	0.004309167	0.00085492	0.004260263	0.002221093	0.001077311	0.002243317	0.001421952	0.001417666	0.002050398	0.001649325	0.000309619	0.000277052	0.000619738	0.002216488
PG(20:0/18:3)-H	0.00065801	0.000161054	0.000807302	0.000273238	0.000194158	0.000334327	0.000225779	0.000295802	0.000340332	0.000258345	0.000046	0.000046	0.000046	0.000386899

Supplemental Table S8: MAG and DAG concentrations (in μM) in EVs isolated from urine collected during the active phase (AP) compared to the inactive phase (IP)

Lipid	AP1	AP2	AP3	AP4	AP5	AP6	AP7	IP1	IP2	IP3	IP4	IP5	IP6	IP7
MAG(18:0)+NH4	0.0272	0.0272	0.52419957	0.927297866	0.0272	0.715417207	1.230171064	1.081571219	0.0272	0.054563128	0.493360504	0.095485285	0.115315564	0.174341768
MAG(18:1)+NH4	0.512440925	0.494167833	0.616902746	0.604180096	0.249100192	1.308864961	1.108857223	2.139240573	1.757242881	0.407644367	0.572924163	0.390416843	0.641381838	0.759218763
MAG(22:6)+NH4	0.656411278	0.684907914	0.91391953	1.448930334	0.23026681	2.373404854	0.626141865	1.510792913	1.891437502	0.746376345	0.172286628	0.080717721	0.560418926	1.097111588
DAG(14:0/20:0)+NH4	0.005	0.005	0.085785358	0.29856755	0.010962341	0.521687014	0.251553275	0.762008112	0.14754176	0.005	0.159206821	0.020526285	0.030282342	0.005
DAG(16:1/18:1)+NH4	0.488046549	0.561917253	2.138728107	1.494762895	0.201950788	3.569963932	2.488411101	5.27105858	1.451469119	0.242671579	1.13004692	0.244485522	0.56456865	0.434640144
DAG(18:0/18:1)+NH4	0.053663734	0.118019247	0.251077326	0.211373935	0.027405853	0.266766868	0.192490052	0.608047515	0.202516024	0.04496409	0.071766714	0.034927662	0.061644197	0.035306106
DAG(18:0/22:6)+NH4	0.132094167	0.148542306	2.500427854	2.797586449	0.050443365	4.812720766	3.439698098	8.239434472	0.406565293	0.092856965	1.817591792	0.101712372	0.239446089	0.243937473
